# Extracellular Galectin-3/Carbohydrate Interactions Modulate Cancer Cellular Migration

**DOI:** 10.3390/ijms27167145

**Published:** 2026-08-09

**Authors:** Mackenzie S. Fricke, Ramat S. Tahir, Hazal K. Ural, Mary J. Cloninger

**Affiliations:** Department of Chemistry and Biochemistry, Montana State University, Bozeman, MT 59717, USA; mackenzie.fricke.24@gmail.com (M.S.F.); ramat.tahir@student.montana.edu (R.S.T.); hkaterinau@gmail.com (H.K.U.)

**Keywords:** galectin-3, dendrimers, glycodendrimers, galectins, cellular migration, cellular viability

## Abstract

Galectin-3-mediated processes are important during many aspects of cancer progression, but they are not well understood. Galectin-3 is present extracellularly, and because of its carbohydrate recognition domain (CRD) and unstructured N-terminal domain (NTD), galectin-3 undergoes multimerization, which influences events such as carbohydrate-controlled cell–cell interactions. Investigations reported herein using an in vitro wound-healing assay show that exogenous galectin-3 inhibits cancer cellular migration. Since the addition of the galectin-3 CRD without the NTD does not arrest cellular migration, we attribute the effect of full-length galectin-3 on migration to the extracellular interactions between multimeric, full-length galectin-3 and extracellular receptors. In this publication, lactose-functionalized dendrimers serve as multivalent binding partners for galectin-3 and are used to mitigate extracellular multivalent galectin/carbohydrate interactions. The addition of lactose-functionalized dendrimers provides significant restoration of cellular migration in the presence of exogenous galectin-3 without increasing cellular viability. Thus, although galectin-3/protein interactions within the cell are known to increase both cellular migration and viability, cell surface galectin-3/carbohydrate interactions have the opposite impact and decrease cellular migration.

## 1. Introduction

**Figure 1:**
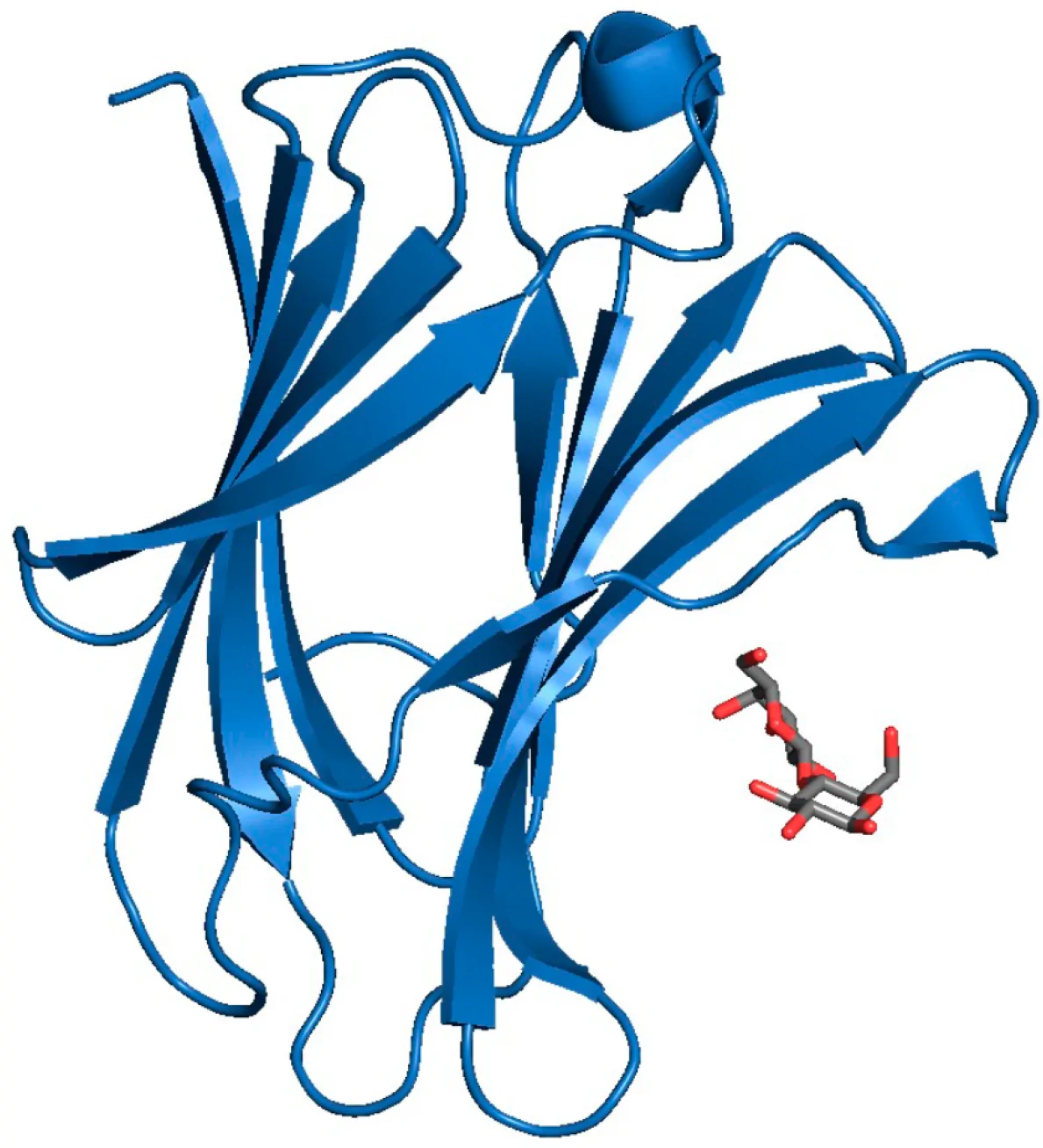
The crystal structure of the carbohydrate recognition domain of galectin-3. PDBID: 3ZSJ.

Galectin-3, like all galectins, is a β-galactoside-binding protein. This 26 kDa protein is the only member of the chimera subfamily of galectins and comprises a disordered, nonhelical collagen-like N-terminal domain and a C-terminal carbohydrate recognition domain (CRD, Figure 1 [1]) [2,3,4]. The N-terminal domain enables aggregation of the galectin-3 proteins into multivalent displays for modulation of carbohydrate binding [5,6,7,8], and the CRD is highly conserved among the known galectins [9]. Galectin-3 is present in the cytoplasm, the nucleus, and extracellularly; extracellular functions of this protein are governed by CRD/carbohydrate binding interactions, while galectin-3/protein binding interactions are responsible for intracellular galectin-3-mediated processes [10,11,12]. Galectin-3 has been implicated in many cancer processes, including tumor formation, cellular migration, angiogenesis, and apoptosis [13,14,15,16].

The role of galectin-3 in cancer cellular migration is especially interesting but is also especially difficult to understand because modulation of cellular migration via both intracellular (galectin-3/protein) and extracellular (galectin-3/carbohydrate) interactions is expected to have significant impacts.

The expression level of galectin-3 has been correlated with cancer cellular migration for a long time and across a variety of cancers, including pancreatic [17,18], gastric [19], breast [20,21], prostate [22], lung [21,23], and ovarian [24] cancers. For example, in pancreatic cell lines, siRNA silencing of galectin-3 expression led to reduced cellular migration but unchanged cellular viability. Decreased expression of matrix metalloproteinase-2 (MMP-2) caused by suppression of the β-catenin/Wnt signaling pathway in the absence of galectin-3 was reported to be responsible for the reduction in cellular migration [25]. Upregulation of galectin-3 expression, on the other hand, resulted in increased cellular migration. For example, overexpression of galectin-3 in miniature pig bone marrow mesenchymal stem cells (BM-MSCs) increased both cellular migration and proliferation. BM-MSC migration was increased primarily due to downregulation of RhoA-GTP activity by galectin-3 [26].

The correlation of galectin-3 expression levels with cancer cellular migratory aptitudes has largely been rationalized based on intracellular galectin-3/protein interactions (vide supra). However, we hypothesized that extracellular galectin-3/carbohydrate interactions must also play an important governing role in cancer cellular migration by controlling how strongly cells are anchored to their current host site and to each other. Indeed, in a study reported by Shetty and coworkers using the HER-2-negative breast cancer cell line MDA MB-231, the interaction at the cell membrane between galectin-3 and galactosylated annexin A2 was shown to impact both cellular viability and migration. Competitive inhibition of this interaction by binding a chickpea lectin (Cicer arietinum agglutinin) downregulated EGFR-mediated signaling, thereby reducing both cancer cell viability and migration [27].

**Figure 2:**
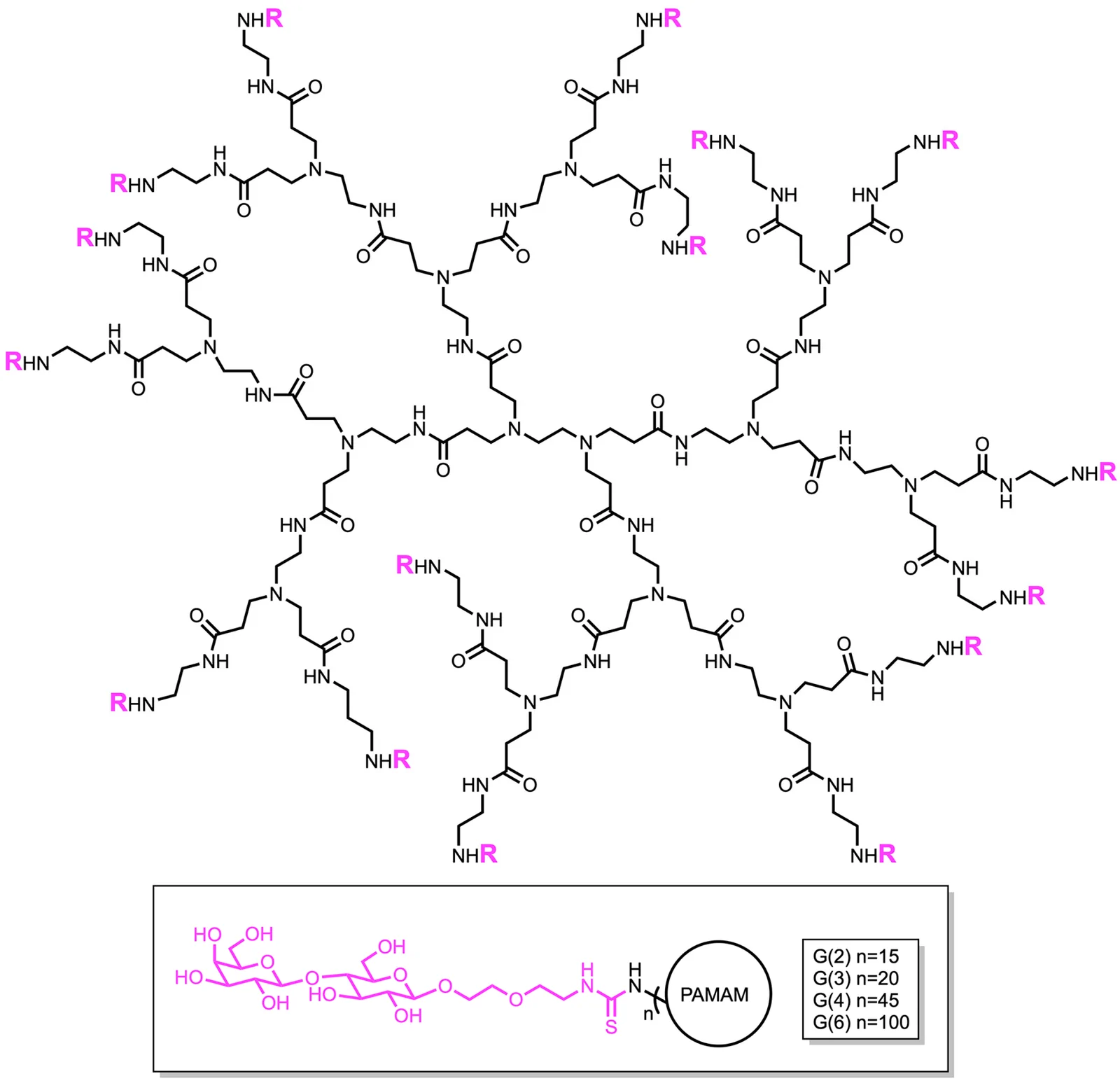
The poly(amidoamine) (PAMAM) dendrimer framework and a schematic of the lactose-functionalized PAMAM dendrimers used in this project (R = lactoside endgroup).

In this manuscript, we describe the use of lactose-functionalized dendrimers to study the effect of extracellular galectin-3/carbohydrate interactions on cancer cellular migration. Dendrimers are highly branched, macromolecular compounds [28,29,30]. The poly(amidoamine) (PAMAM) dendrimers (Figure 2) used in this study have a theoretical doubling of the number of terminal amino endgroups with each successive generation (G(0) has four amino termini, G(1) has eight amino termini, etc.) and were functionalized with an average of fifteen (G(2)), twenty (G(3)), forty-five (G(4)) and one hundred (G(6)) lactosides (Figure 2) [31]. We, and others, have found that lactose-functionalized dendrimers are important tools for studying galectin-mediated cellular processes [31,32,33,34,35,36,37,38,39,40,41,42].

Indeed, carbohydrate-functionalized synthetic multivalent frameworks have been used in several important studies aimed at elucidating the structure and function of galectins. The use of glycodendrimers, linear glycopolymers, glycopolypeptides and glycoproteins, glyconanoparticle rods and fibers, and amphiphilic glycosystems to study galectins has recently been reviewed [43]. Noteworthy examples since the publication of this review describing the use of carbohydrate-functionalized synthetic multivalent frameworks to improve understanding of the roles of galectins in cancer migration include a study by Callmann and coworkers. In this work, galactose-functionalized linear polymers synthesized via ring-opening metathesis polymerization (ROMP) were shown to reduce both cellular viability and migration in 4T1 triple-negative breast cancer (TNBC) cells [44]. A second timely report describes the use of a calcium phosphate nanoparticle functionalized with modified citrus pectin such that it could capture serum galectin-3. The nanoparticle was also loaded with anti-apoptotic paris saponin VII (PSVII) and siGalectin-3. The galectin-3 surface binding enabled these nanoparticles to target drug-resistant colon cancer cells with elevated integrin avβ3 expression. The siGalectin-3 decreased galectin-3 expression levels in the cancer cells; simultaneous delivery of siGalectin-3 and PSVII suppressed cancer cell proliferation and metastasis in a mouse model [45].

Here, we report that lactose-functionalized glycodendrimers restore in vitro cancer cellular migration in the presence of migration-arresting exogenous galectin-3. Glycodendrimers effectively restore cancer cellular migration but do not by themselves reduce or accelerate the rate of cancer cellular viability. When added with galectin-3, the inherently nontoxic glycodendrimers caused a reduction in cellular viability in addition to causing an increase in cellular migration. Generally, as described above, reductions in migration tied to galectin-3-mediated processes have also resulted in reductions in cellular viability, and these effects have been attributed to alterations in intracellular signaling processes. Increases in both cellular migration and viability have likewise been attributed to changes caused by intracellular galectin/protein interactions.

This report provides compelling evidence for the importance of extracellular galectin-3/carbohydrate interactions in the modulation of cancer cellular migration. Thus, in addition to its well-documented intracellular pathways for modulation of cellular migration, galectin-3 also controls cellular migration by attenuating cellular adhesion via extracellular protein–carbohydrate interactions.

## 2. Results

### 2.1. Scratch Assay

A scratch assay with lactose-functionalized dendrimers was used to assess the impact of both full-length galectin-3 and the CRD on cancer cell migration. Two cancer cell lines, DU 145 prostate and HT-1080 fibrosarcoma, were selected for this investigation based on their relative endogenous production of galectin-3. DU 145 has a high amount of galectin-3, while the amount of endogenous galectin-3 in HT-1080 is much lower [6]. A high level of galectin-3 indicates a strong likelihood of existing galectin-3-mediated processes, and for this reason we hypothesized that the impacts of excess galectin-3 would be more prominent in DU 145 cells and less impactful in HT-1080 cells.

#### 2.1.1. Scratch Assay with DU 145 Cancer Cells and Lactose-Functionalized Dendrimers

**Figure 3:**
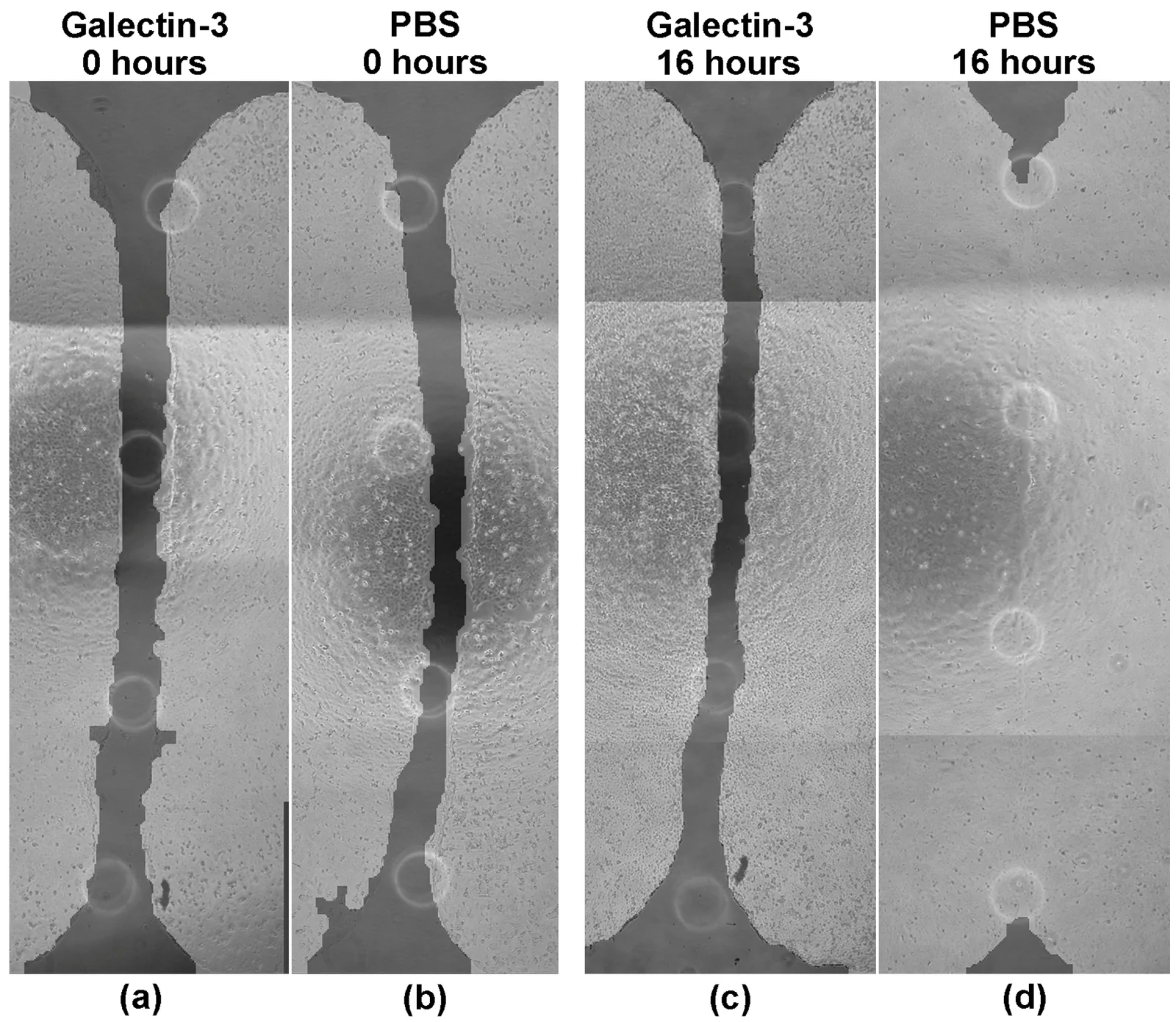
DU 145 prostate carcinoma cell migration into the scratch (**a**) in the presence of galectin-3 at the start of the assay (0 h), (**b**) in buffer (PBS) lacking galectin-3 at the start of the assay (0 h), (**c**) in the presence of galectin-3 at the last time point in the assay (16 h), and (**d**) in buffer (PBS) without galectin-3 at the last time point in the assay (16 h).

A scratch assay was designed to investigate the effect of lactose-functionalized dendrimers and exogenous galectin-3 on the migration of DU 145 cells, which are prostate carcinomas that contain a relatively high concentration of endogenously produced galectin-3 [46]. A scratch in the cell population was created using a two-well insert (ibidi), and the extent of migration into the scratch was recorded every 4 h for a total of 16 h. Micrographs from the beginning and ending points of this assay are shown in Figure 3, and the full sets of micrographs for galectin-3 and PBS are shown in Figures S1 and S2 in the Supplementary Information. Cell migration into the scratch at each subsequent time point was normalized to the cell-free area at the initial time point. The migration rate for the cells was determined by adding PBS devoid of any additives. The migration rate upon addition of galectin-3 or galectin-3 with glycodendrimers was then compared to the degree of migration into the scratch at each time point when migration is unaltered (PBS). Glycodendrimer concentrations were used such that the concentration of lactose was approximately 1 mM for all experiments, while the concentration of galectin-3 was kept constant at 7.4 μM for all experiments.

**Figure 4:**
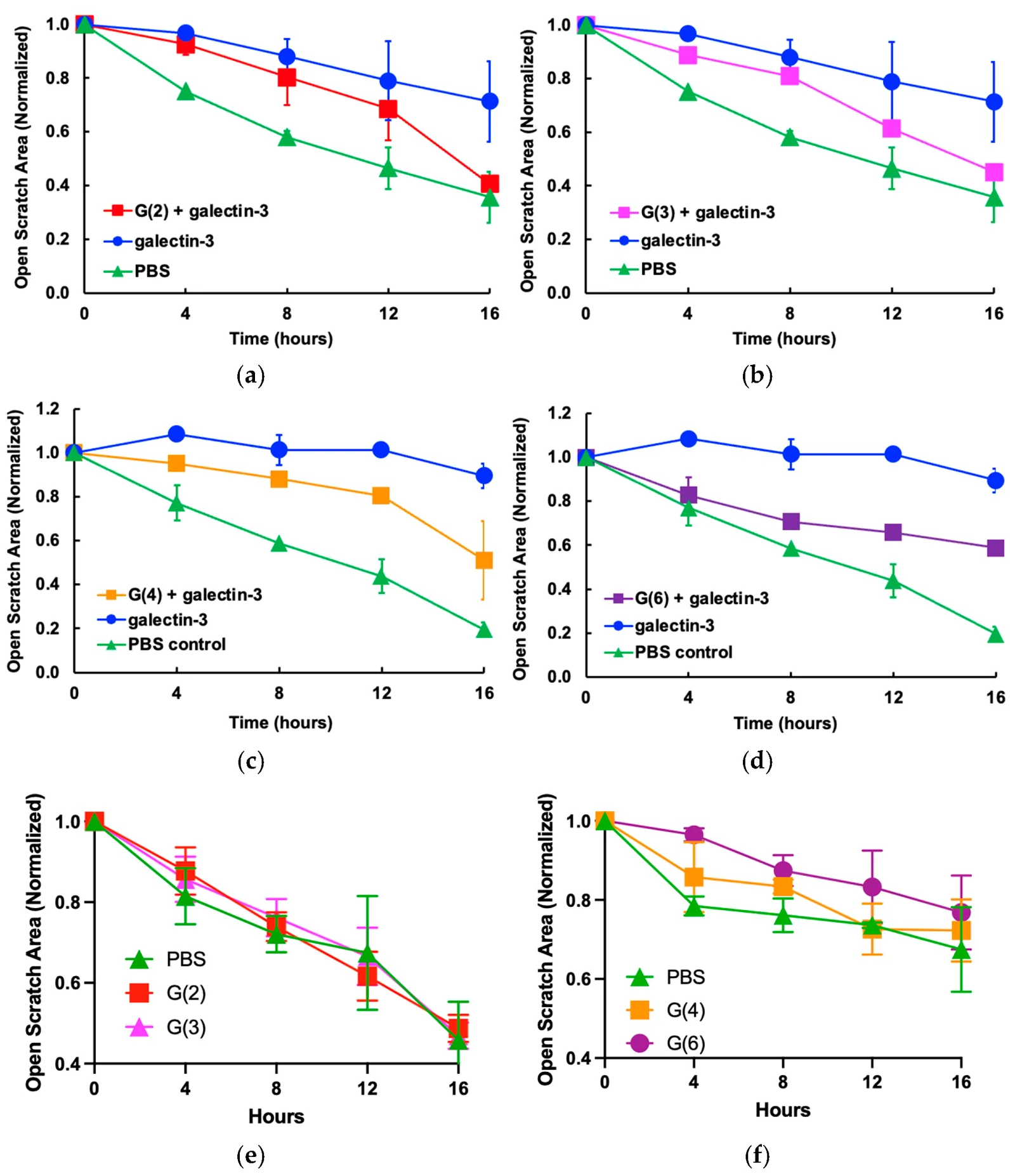
DU 145 prostate carcinoma cell migration into the scratch in the presence of PBS (green triangles), exogenous galectin-3 (blue circles) and each generation of lactose-functionalized dendrimer: (**a**) lactose-functionalized G(2) dendrimer and galectin-3 (red squares), (**b**) lactose-functionalized G(3) dendrimer and galectin-3 (pink squares), (**c**) lactose-functionalized G(4) dendrimer and galectin-3 (orange squares), (**d**) lactose-functionalized G(6) dendrimer and galectin-3 (purple squares), (**e**) control experiment with lactose-functionalized G(2) (red squares) and G(3) (pink triangles) dendrimers in the absence of galectin-3, (**f**) control experiment with lactose-functionalized G(4) (orange squares) and G(6) (purple circles) dendrimers in the absence of galectin-3. The assay was run for 16 h, and scratch areas were recorded by image acquisition every four hours and normalized to the open scratch area at 0 h. Error bars show the standard deviation for triplicate repeats of the experiment.

When a solution of galectin-3 in PBS was added to the well containing a scratch, the migration of the cells into the scratch was significantly decreased (Figure 3c and Figure 4, blue circles) compared to the degree of cellular migration that is observed when PBS lacking galectin-3 was added instead (Figure 3d and Figure 4, green triangles). When G(2) and G(3) lactose-functionalized dendrimers were added with galectin-3, migration was initially retarded relative to the PBS control. However, after 16 h, lactose-functionalized G(2) and G(3) dendrimers exhibited a migration trend similar to the migration that occurred when only PBS was added (Figure 4, red for G(2) and pink for G(3)). G(2) and G(3) dendrimers are relatively small, have fewer lactoside endgroups than the other glycodendrimers that were tested, and are somewhat amorphous in shape. The observation of similar results for these two glycodendrimers is therefore not surprising.

When the scratch contained lactose-functionalized G(4) or G(6) dendrimers and galectin-3, the scratch closure was neither as slow as the galectin-3 control nor as fast as the PBS control at all time points (Figure 4, orange for G(4) and purple for G(6)). Because G(4) and G(6) glycodendrimers are more spherical and bear more lactosides than G(2) and G(3), the observation of similar results for these two glycodendrimers is also reasonable. As shown in Figure 4, all four generations of glycodendrimers prevent exogenous galectin-3 from fully arresting cellular migration. The addition of glycodendrimers with galectin-3 restores cellular migration either fully (G(2) and G(3)) or substantially (G(4) and G(6). The micrographs of the data are provided in Figures S3–S6 in the Supplementary Information. Control experiments in which each of the glycodendrimers was added without the addition of galectin-3 show that the addition of the glycodendrimers alone does not impede cellular migration into the scratch (Figure 4e,f and Figures S7–S11 in the Supplementary Information).

#### 2.1.2. Scratch Assay with HT-1080 Cancer Cells and Lactose-Functionalized Dendrimers

**Figure 5:**
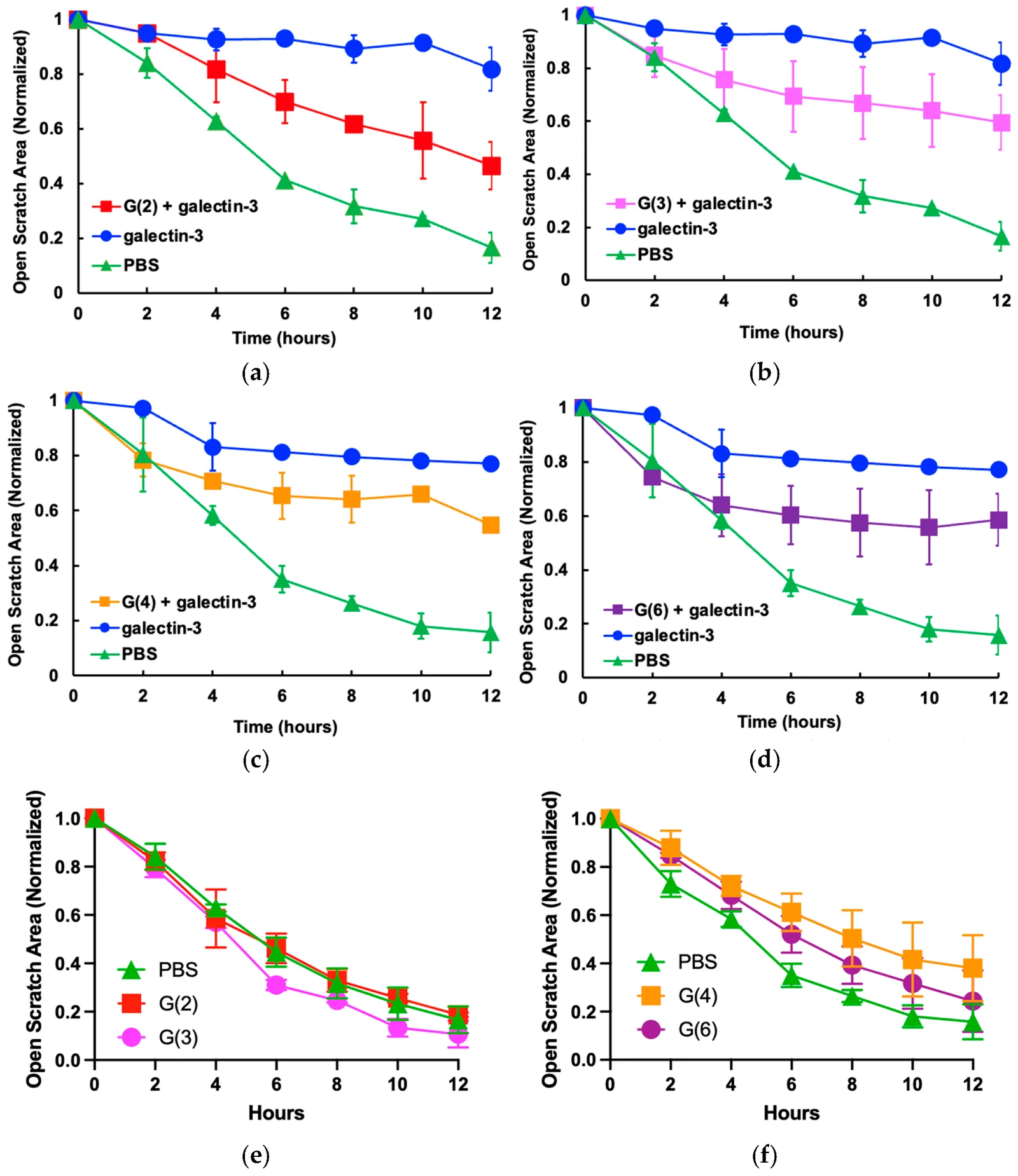
HT-1080 human fibrosarcoma cellular migration rate in the presence of PBS (green triangles), exogenous galectin-3 (blue circles), and each generation of lactose-functionalized dendrimers: (**a**) lactose-functionalized G(2) dendrimer and galectin-3 (red squares), (**b**) lactose-functionalized G(3) dendrimer and galectin-3 (pink squares), (**c**) lactose-functionalized G(4) dendrimer and galectin-3 (orange squares), (**d**) lactose-functionalized G(6) dendrimer and galectin-3 (purple squares), (**e**) control experiment with lactose-functionalized G(2) (red squares) and G(3) (pink circles) dendrimers in the absence of galectin-3, (**f**) control experiment with lactose-functionalized G(4) (orange squares) and G(6) (purple circles) dendrimers in the absence of galectin-3. The assay was run for 12 h, and scratch areas were recorded by image acquisition every two hours and normalized to the open scratch area at 0 h. Error bars show the standard deviation for triplicate repeats of the experiment.

The scratch assay was repeated with HT-1080 fibrosarcoma cells [47], which produce low levels of galectin-3. Since this cell line migrated much more quickly than the previous one, the assay was adjusted to be monitored every two hours for twelve hours. As reported using DU 145 cells, adding exogenous galectin-3 in PBS to the scratch resulted in significantly decreased migration into the scratch (Figure 5, blue circles). Adding an identical amount of the buffer (PBS) resulted in rapid scratch closure during the twelve-hour assay (Figure 5, green triangles).

When the dendrimers were added with the galectin-3 (Figure 5a–d, red, pink, orange, and purple squares), some restoration of migration was observed. Lactose-functionalized G(2) and G(3) dendrimers did not achieve nearly the same restoration of migration in HT-1080 cell lines as was observed for the DU 145 cell line, but some recovery was achieved. A small but significant recovery of migration was also achieved for lactose-functionalized G(4) and G(6) dendrimers. Micrographs for galectin-3, PBS, and lactose-functionalized dendrimers with galectin-3 are shown in Figures S12–S17 in the Supplementary Information. When lactose-functionalized dendrimers are added to the scratch without adding galectin-3, cellular migration is generally comparable to that of cells without any additives. The graphs of migration for glycodendrimers in the absence of galectin-3 are shown in Figure 5e,f and Figure S18, and micrographs are shown in Figures S19–S22 of the Supplementary Information.

**Figure 6:**
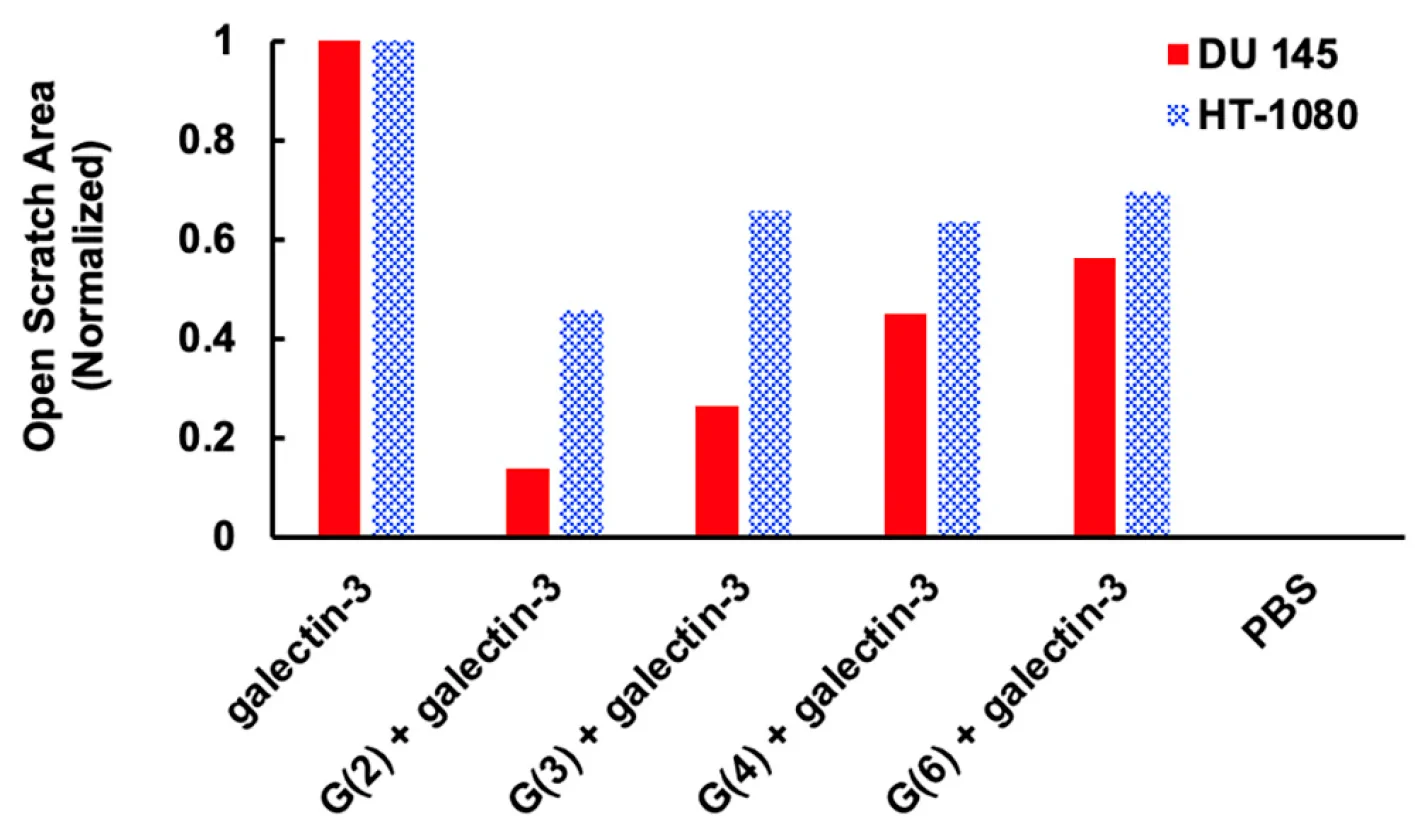
Comparison of the open scratch areas for DU 145 (red bars) and HT-1080 (blue patterned bars) cells at the scratch assay endpoint.

Figure 6 shows a comparison between the two cell lines for the percentage of open scratch area at the assay endpoints of 16 h (DU 145) and 12 h (HT-1080). The glycodendrimers restore the galectin-3-inhibited migration of DU 145 cells more than they restore galectin-3-inhibited migration of HT-1080 cells, which correlates well with the amount of galectin-3 present in the cell lines.

#### 2.1.3. Scratch Assay with Full-Length Galectin-3 and the Galectin-3 CRD

**Figure 7:**
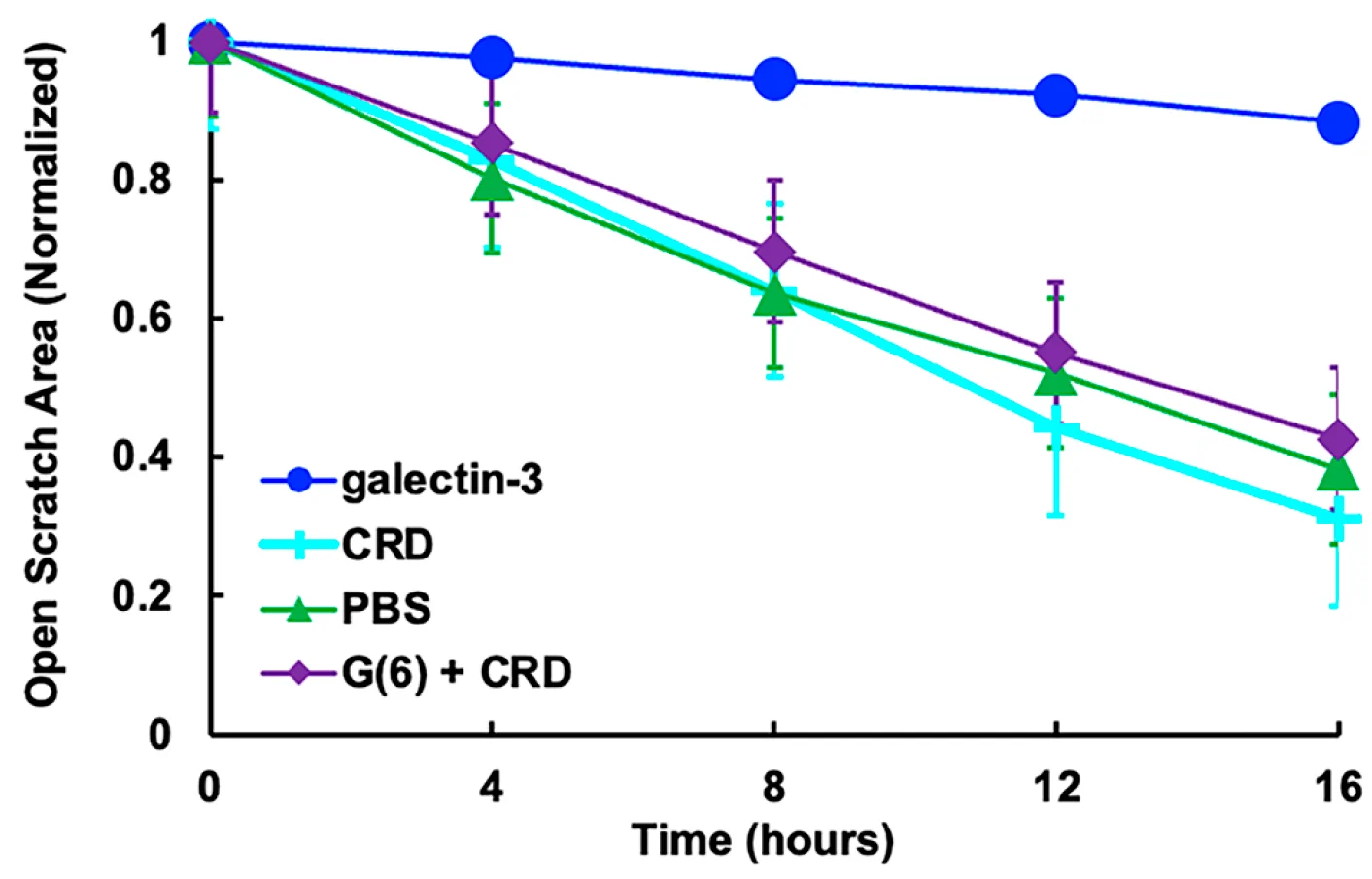
DU 145 prostate carcinoma cellular migration in the presence of full-length galectin-3 (blue circles), the galectin-3 CRD (cyan crosses), lactose-functionalized G(6) dendrimer and CRD (purple diamonds), and PBS control (green triangles). The assay was run for 16 h, and scratch areas were recorded by image acquisition every four hours and normalized to the open scratch area at 0 h. Error bars show the standard deviation for triplicate repeats of the experiment except for CRD, which was a duplicate experiment.

The CRD of the galectins binds β-galactosides, and the disordered N-terminal domain of galectin-3 contains the function of self-association. Our next investigation was to determine if the N-terminal domain had an impact on the observed migration result. Thus, the CRD (amino acids 112–250) was added to DU 145 scratches and compared to scratches in the presence of the full-length galectin-3. As shown in Figure 7, the CRD was unable to impact the migration of DU 145 cells in the same manner as full-length galectin-3. Cellular migration in the presence of the CRD (in PBS) was the same as the migration observed for the cells in the presence of pure PBS (Figure 7, cyan crosses and green triangles; micrographs are shown in Figure S23 in the Supplementary Information). Moreover, when a lactose-functionalized G(6) dendrimer was added with the CRD in the scratch (Figure 7, purple diamonds; micrographs are shown in Figure S24 in the Supplementary Information), no significant change was observed from the PBS control. This is quite different from the result with full-length galectin-3: cells incubated with lactose-functionalized G(6) dendrimer in the presence of full-length galectin-3 (Figure 4, purple squares) showed significantly inhibited migration when compared to the migration of cells incubated in PBS alone. The fact that the CRD, CRD plus lactose-functionalized G(6) dendrimer, and PBS control are indistinguishable highlights the importance of the galectin-3 N-terminal domain in mediating extracellular anchoring and migration processes.

#### 2.1.4. Scratch Assay with Monomeric Lactose to Study Multivalent Effects

Experiments were performed to evaluate the multivalent effect of glycodendrimers in this assay. Although each monovalent galectin-3/carbohydrate binding interaction occurs with relatively weak affinity (e.g., K_D_ = 56 ± 8 μM for lactose [48] and K_D_ = 42 ± 2 μM for isopropyl thiogalactoside (IPTG) [49]), multivalent protein–carbohydrate interactions afford synergistic enhancements in binding and are often implicated in cellular recognition processes. In general, multivalent effects are defined as synergistic enhancements that can occur when multiple ligands are simultaneously bound by multiple receptor binding sites. Multivalent effects are important for a wide range of interactions at the cell surface, including the bacterial and viral invasion of cells, the mounting of an immune response, and tumor formation [50,51,52,53].

**Figure 8:**
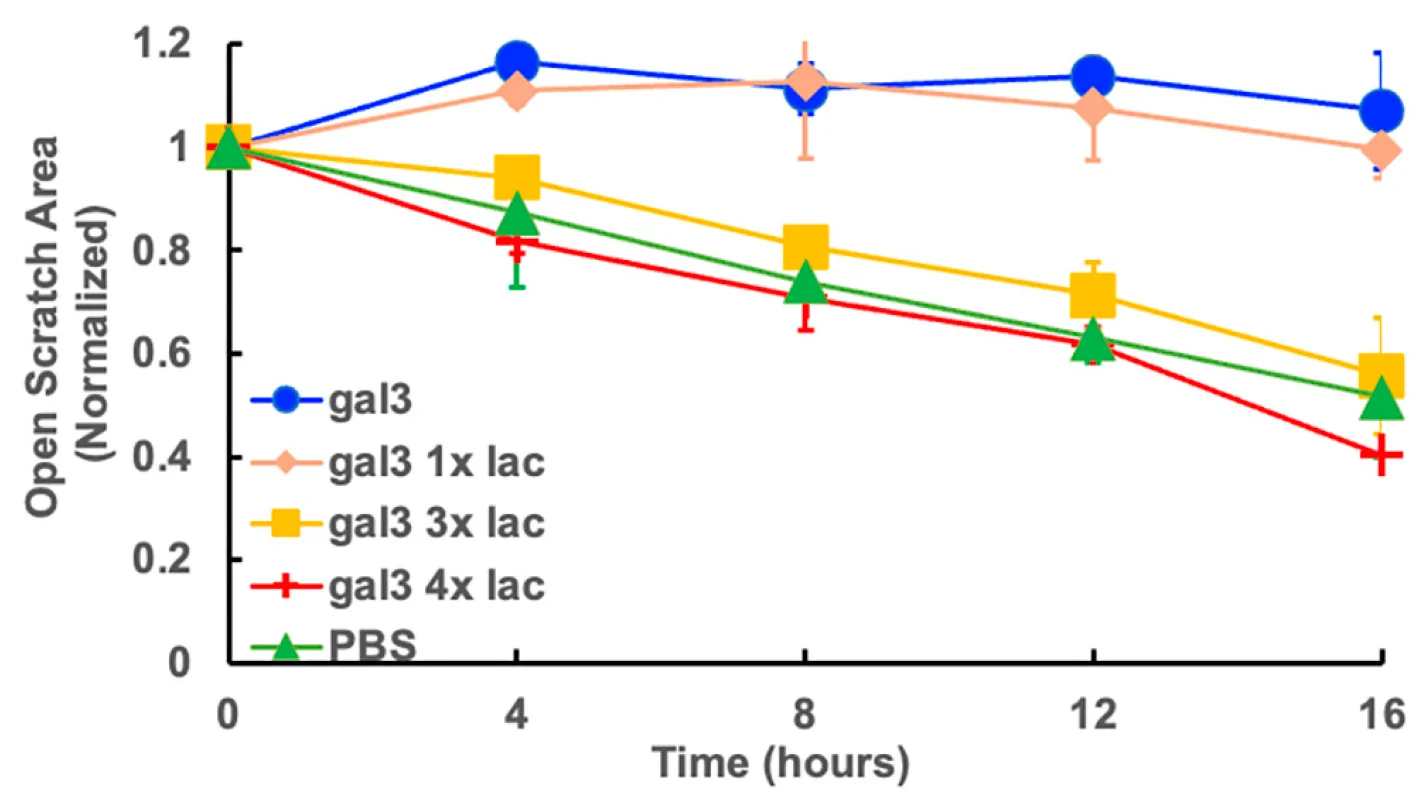
Migration of DU 145 cells with added galectin-3 and increasing concentrations of monomeric lactose. Peach diamonds: 850 µM lactose; gold squares: 2.54 mM lactose; red crosses: 3.39 mM lactose; blue circles, galectin-3 alone; green triangles, PBS control. The assay was run for 16 h, and scratch areas were recorded by image acquisition every four hours and normalized to the open scratch area at 0 h. Error bars show the standard deviation for triplicate repeats of the experiment.

When monomeric lactose was added with exogenous galectin-3, twice as much monomeric lactose as the concentration of lactose in the lactose-functionalized dendrimer experiments was required to achieve the same effect on migration that was observed for the PBS control (Figure 8, gold squares). No observable change in migration compared to the galectin-3 control occurred when monomeric lactose was added at approximately the same concentration as the lactosides present in the G(4) glycodendrimer assay (Figure 8, peach diamonds; lactose concentration is 850 µM; G(4) was 927 µM in lactosides). Micrographs are shown in Figures S25–S27 in the Supplementary Information.

### 2.2. Fluorescence Imaging Results

**Figure 9:**
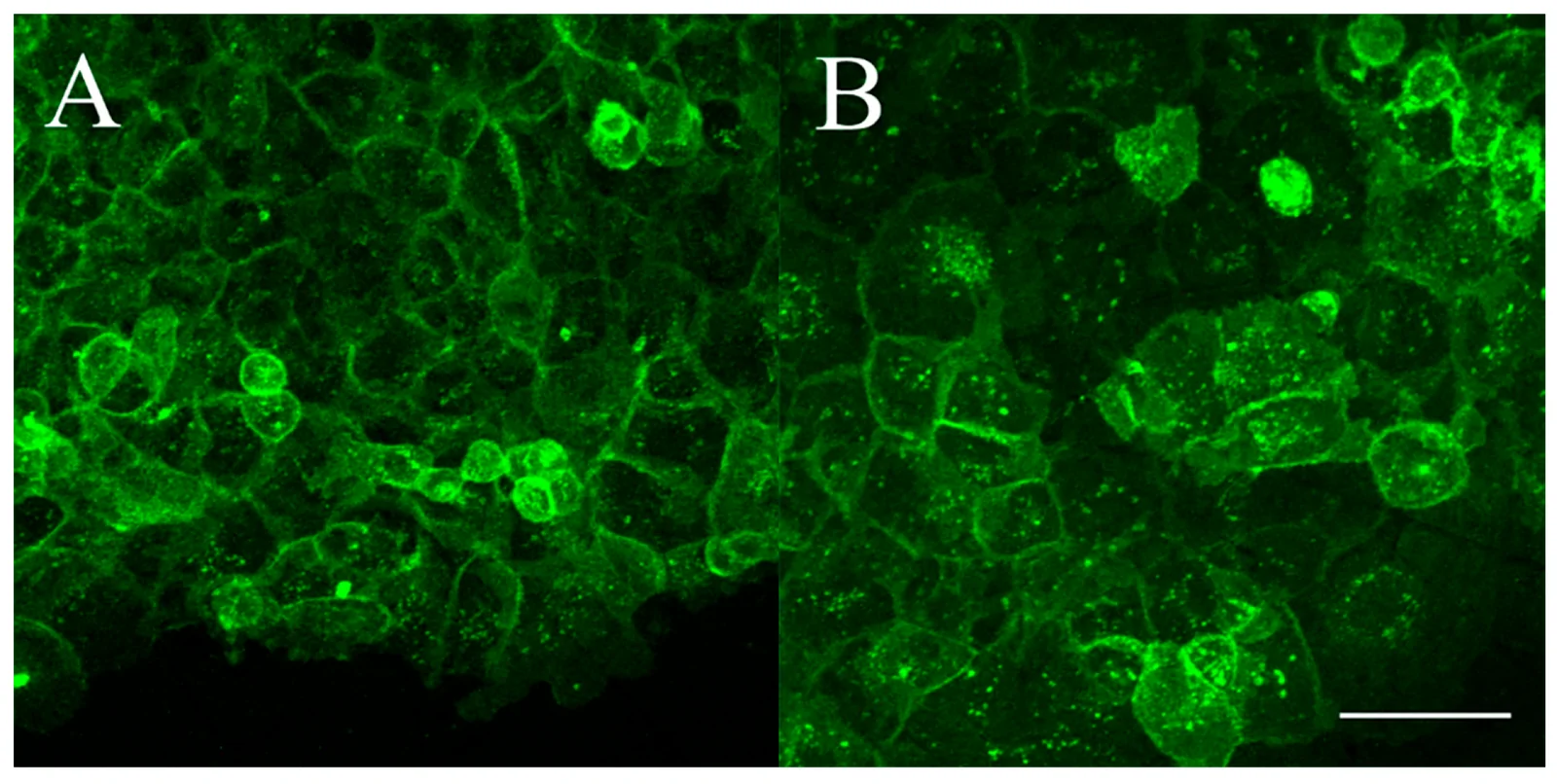
Anti-galectin-3-Alexa Fluor 488 labeling of DU 145 cells in migration assay conditions with exogenous galectin-3 after migration for (**A**) 4 h and (**B**) 12 h. Scale bar is 50 μm.

To visualize the localization of galectin-3 during the scratch assay, immunofluorescence was performed. Alexa Fluor 488-conjugated galectin-3 antibody was added at 4 or 12 h in the scratch assay using DU 145 cells, and the scratch was fixed and mounted after 1 h of incubation. The samples were visualized using confocal laser scanning microscopy (CLSM). Figure 9 shows that when galectin-3 was added to the scratch, the smooth webbing-like pattern of galectin-3 in the intercellular area is clearly visible. The black portion at the bottom of Figure 9A (4 h) and in the lower right quadrant of Figure 9B (12 h) shows the border between the leading edge of cells and the remaining open space from the scratch. Although the overall fluorescence is the same for images taken at 4 h and 12 h (values are provided in Table S12 in the Supplementary Information), small, spotted features indicating aggregates of galectin-3 are more prevalent at 12 h (Figure 9B) than at 4 h (Figure 9A).

**Figure 10:**
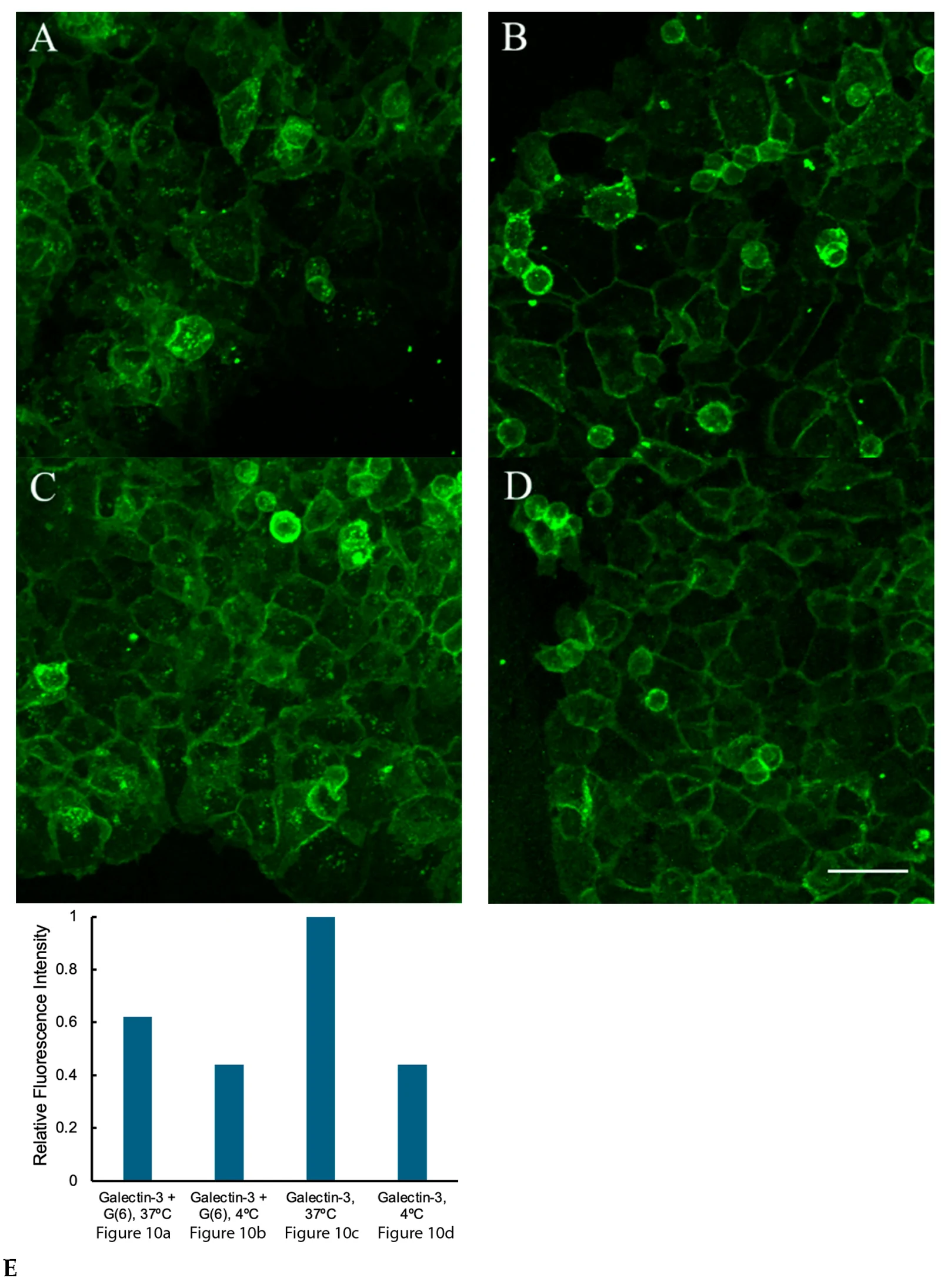
Scratch Assay using DU 145 cells for 4 h followed by antibody incubation at 37 °C or 4 °C. (**A**) Galectin-3 and lactose-functionalized G(6) dendrimers with antibody binding at 37 °C, (**B**) galectin-3 and lactose-functionalized G(6) dendrimers with antibody binding at 4 °C. (**C**) galectin-3 with antibody binding at 37 °C, and (**D**) galectin-3 with antibody binding at 4 °C, and (**E**) graph of the relative fluorescence intensities from images in (**A**–**D**). Scale bar 50 μm.

To determine whether the amount of exogenous galectin-3 that adheres to the scratch changes in the presence of glycodendrimer, the fluorescence when galectin-3 was added with lactose-functionalized G(6)-PAMAM (Figure 10A) was compared to the fluorescence intensity when only galectin-3 was added. Figure 10A shows the result when Alexa Fluor 488-conjugated galectin-3 antibody was added at 4 h to the scratch assay for galectin-3 plus lactose-functionalized G(6)-PAMAM, with the open scratch shown as the black area in the lower right quadrant. The fluorescence intensity of this sample is reduced by approximately 40% relative to the fluorescence intensity when only galectin-3 is added (Figure 10C). The same assay was repeated with antibody binding conducted at 4 °C to inhibit endocytosis (Figure 10B shows galectin-3 plus lactose-functionalized G(6)-PAMAM at 4 °C; Figure 10D shows galectin-3 alone at 4 °C). The fluorescence intensity drops by nearly 30% in Figure 10B relative to Figure 10A, indicating that nearly 30% of the galectin-3 was endocytosed in the presence of lactose-functionalized G(6)-PAMAM. When only galectin-3 was added (without glycodendrimer), a comparison of antibody binding at 37 °C and at 4 °C shows a reduction in fluorescence of 55%. This indicates that approximately 55% of the galectin-3 is endocytosed when glycodendrimers are not present. Webbing is still observed in both samples at 4 °C, but the number of bright, punctate spots is reduced, suggesting that these spots of aggregated galectin-3 arise, at least in part, upon endocytosis. The differences in fluorescence intensity are summarized in Figure 10E, with values provided in Table S13 in the Supplementary Information.

### 2.3. Cellular Viability Assay

A significant increase in cellular viability could cause the scratch in the wound-healing assay to fill with cells simply because the abundance of cells would need a place to adhere. Thus, without an independent evaluation of cellular viability, an increase in viability could mistakenly be attributed to an increase in migration (or a decrease in migration could be attributed to a decrease in viability). If both cellular viability and migration increased, then both would logically be expected to contribute toward closure of the scratch. To accurately interpret the restoration of scratch closure by lactose-functionalized dendrimers in the presence of galectin-3 (Figure 4 and Figure 5), we therefore needed to perform cellular viability assays. For the cellular viability assay, the amount of ATP was quantified using a luciferase-based bioluminescence assay. Wells in a 96-well plate were seeded with cells 24 h prior to addition of lactose-functionalized dendrimers, galectin-3, the CRD, or combinations of lactose-functionalized dendrimers and galectin-3. Because the additives were dissolved in PBS, control experiments using PBS without any additives (positive) and using 0.5% SDS (negative) were used. After incubating for 72 h, CellTiter-Glo (CTG) reagent (Promega) was added to simultaneously lyse the cells and detect ATP.

#### 2.3.1. Cellular Viability Assay with Galectin-3, the CRD, and Glycodendrimers

**Figure 11:**
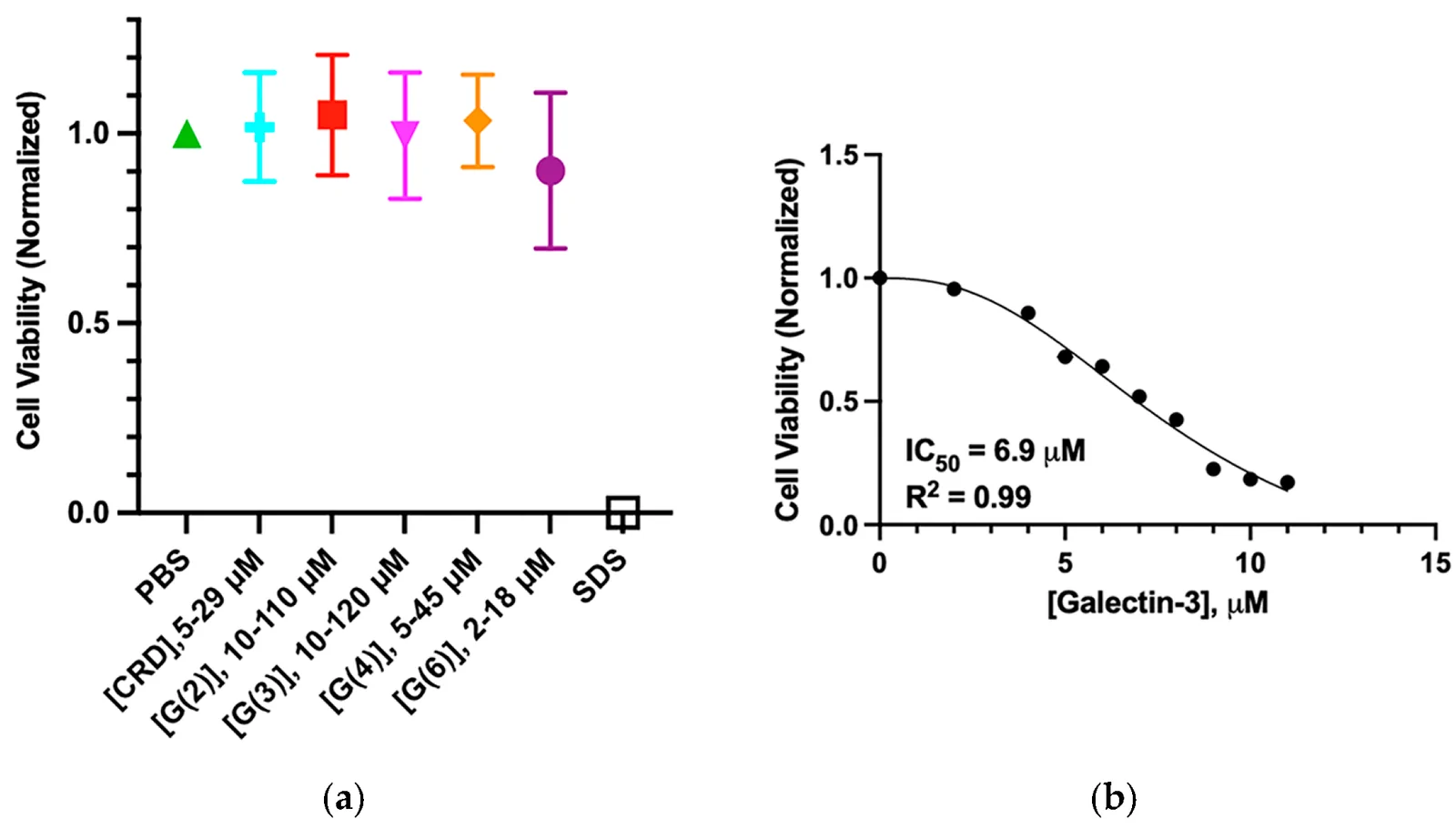
(**a**) Cellular viability results with PBS (upward pointing green triangle, positive control), CRD (cyan cross), lactose-functionalized G(2)-PAMAM (red square), lactose-functionalized G(3)-PAMAM (downward pointing pink triangle), lactose-functionalized G(4)-PAMAM (orange diamond), lactose-functionalized G(6)-PAMAM (purple circle), and 0.5% SDS (open square). (**b**) The IC_50_ value for galectin-3 in DU 145 cells. Error bars represent a minimum of triplicate experiments for all except the CRD, which was a duplicate experiment.

As shown in Figure 11a, the CRD and all four lactose-functionalized dendrimers are nontoxic across a large concentration range with DU 145 cancer cells. The cellular viability is the same when PBS is added with or without the additives. SDS was used as a negative control and is included in Figure 11a.

The IC_50_ value for galectin-3 in DU 145 cells was determined to be 6.94 μM as shown in Figure 11b. The 95% confidence interval values for this IC_50_ are 6.56 and 7.33. Thus, for galectin-3, reductions in both cellular viability and migration were observed.

#### 2.3.2. Cellular Viability Assay When Galectin-3 and Glycodendrimers Are Co-Administered

**Figure 12:**
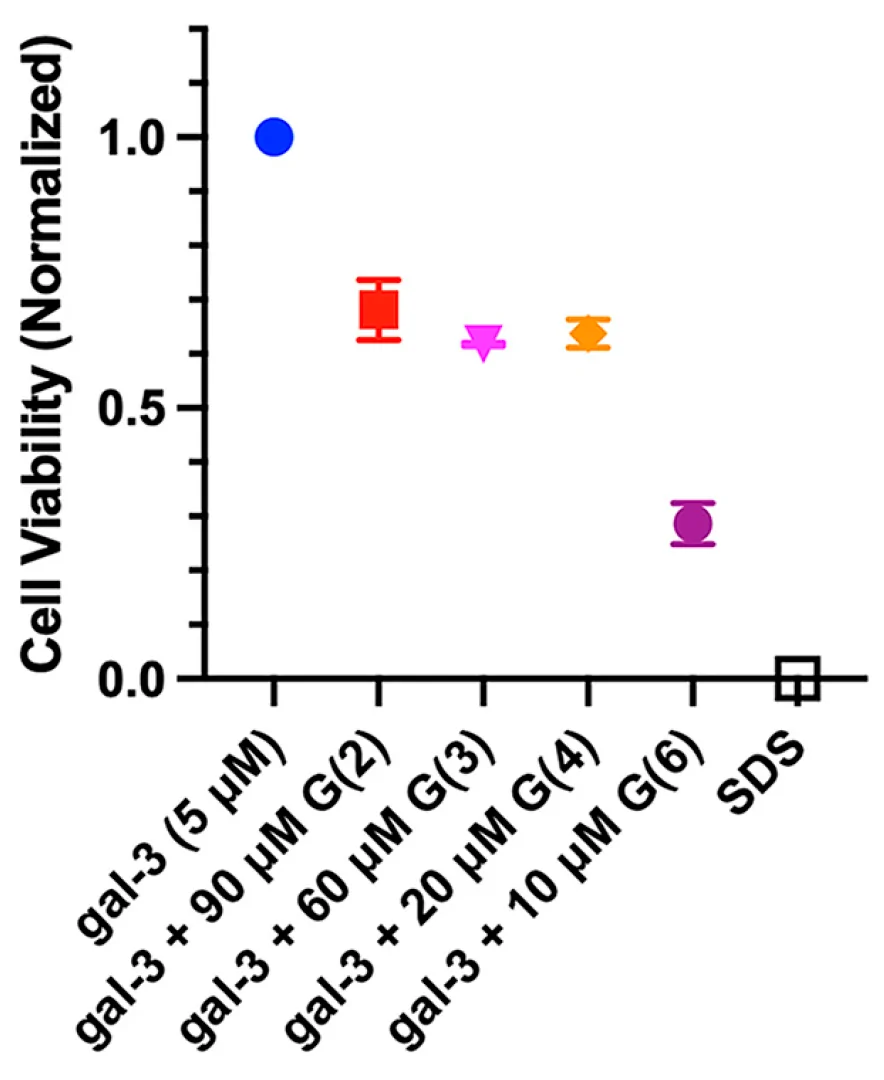
Cellular viability results with 5 μM galectin-3 (blue circle), and 5 μM galectin-3 plus lactose-functionalized G(2)-PAMAM (red square), plus lactose-functionalized G(3)-PAMAM (downward pointing pink triangle), plus lactose-functionalized G(4)-PAMAM (orange diamond), or plus lactose-functionalized G(6)-PAMAM (purple circle), and 0.5% SDS (open square). Concentrations of dendrimers are reported on a per dendrimer basis, viability with galectin-3 is normalized to 1, and error bars show the standard deviation for at least triplicate repeats of the experiment.

Compared to the results with galectin-3 or the glycodendrimers alone (Figure 11), cellular viability is reduced by an additional 35% when glycodendrimers are co-administered with galectin-3, as shown in Figure 12, with the cellular viability in the presence of galectin-3 normalized to 1 for clarity. Thus, when migration is increased by coadministration of glycodendrimers and galectin-3 relative to administration of galectin-3 alone, cellular viability is simultaneously reduced (Figure 12 compared to Figure 4). Tables S1–S11 in the Supplementary Information show the complete data sets that are summarized in Figure 11 and Figure 12.

## 3. Discussion

Galectin-3 has been implicated in many cancer pathways [16,32]. One of the most devastating diagnostically is metastasis, which is a multistep process in which tumor cells migrate to a distant site and form a secondary tumor [54]. In order to metastasize, a tumor must disrupt its cell–matrix interactions and invade a nearby circulatory system, usually by increasing the cell–cell adhesions [55]. Since galectin-3 can impact cell–cell adhesion and cell–matrix interactions [10,56], extracellular galectin-3 is expected to have a profound impact on the migratory propensity of cancer cells. Indeed, when galectin-3 is added to the cancer cells, cellular migration is inhibited (Figure 3, Figure 4 and Figure 5). Crosslinking of the cancer cells by galectin-3 serves to anchor them in their place, preventing migration.

The NTD of galectin-3 (amino acids 1–111) is required for homotypic cellular aggregation; the CRD, which is a truncated variant consisting only of the carbohydrate recognition domain (amino acids 112–250) does not induce homotypic cellular aggregation comparable to the results with full-length galectin-3 (amino acids 1–250). Crosslinking of cancer cells by galectin-3 has been attributed to the ability of the galectin-3 NTDs to intertwine, which results in the multivalent presentation of carbohydrate binding receptor domains. Similarly, when the NTD is not present, the CRD does not inhibit cellular migration (Figure 7) and does not reduce cellular viability (Figure 11a) like full-length galectin-3 does (Figure 3 and Figure 11b). Crosslinking of the cells, which would be expected to anchor them and prevent migration, can only occur when the NTD is present to create a multivalent carbohydrate receptor. Multivalent galectin-3/carbohydrate interactions at the cell surfaces enable sufficiently strong intercellular crosslinking to prevent cellular migration.

**Figure 13:**
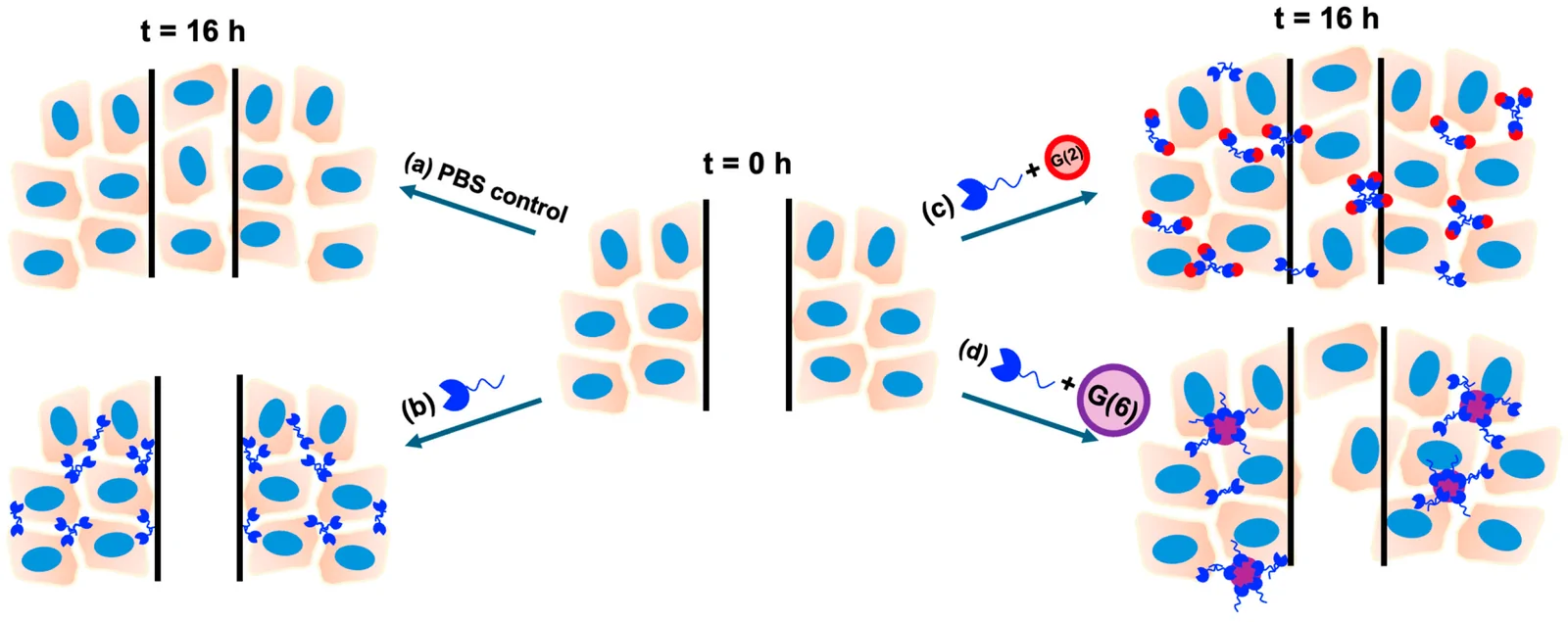
(**a**) When only PBS is added, cells migrate fully into the scratch. (**b**) Galectin-3 crosslinking of cells anchors them in place, preventing migration. (**c**) Small lactose-functionalized dendrimers (G(2) and G(3)) bind galectin-3 and divert the protein from cellular crosslinking, restoring migration. (**d**) Large lactose-functionalized dendrimers (G(4) and G(6)) bind galectin-3 to make larger clusters that partially divert galectin-3 but also partially maintain cellular anchoring, resulting in partially restored cellular migration.

The addition of glycodendrimers with galectin-3 restores cellular migration either fully (G(2) and G(3)) or substantially (G(4) and G(6)). As depicted pictorially in Figure 13, lactose-functionalized dendrimers become competitive binding partners for galectin-3 that can bind up some of the galectin-3. The most likely reason for the observed result with the small glycodendrimers is shown schematically in Figure 13c. Lactose-functionalized G(2)- and G(3)-PAMAMs are able to efficiently bind galectin-3 to create small galectin-3/glycodendrimer clusters that sequester galectin-3 and prevent galectin-3-mediated crosslinking of the cancer cells. Lactose-functionalized G(4)- and G(6)-PAMAMs have been shown to nucleate the formation of large clusters of galectin-3 [31], which are likely to partially crosslink and anchor the cells in place. Thus, as shown in Figure 13d, not all of the galectin-3 is prevented from interacting with carbohydrates on the cell surfaces when the larger generations of lactose-functionalized dendrimers are used. The generation-dependent mechanisms are consistent with our prior work. Small lactose-functionalized G(2) dendrimers were previously shown to provide competitive binding sites for galectin-3 in solution, thereby inhibiting galectin-3/MUC1-mediated cancer cellular aggregation, while large G(6) dendrimers provided sufficient lactose valency to crosslink cell surface galectin-3 and increase cellular aggregation [6,7]. The generation-dependent restoration of migration observed in this study is therefore consistent with a shift from predominantly solution-phase competitive binding for smaller dendrimers to increasing membrane-phase binding for larger generations. In the control experiments with monomeric lactose, lactose could effectively prevent galectin-3 from binding to the cells, but the concentration of lactose required to restore cellular migration was 2-fold higher than the concentration of lactosides present when the dendrimers were added (2.5 mM lactose vs. approximately 1 mM lactose endgroups for the dendrimers). Because monovalent lactose binding to galectin-3 is weaker than the multivalent glycodendrimer/galectin-3 interactions, approximately 2-fold more monovalent lactose was required to divert galectin-3 from binding to cell surface carbohydrates and crosslinking cells such that cellular migration was restored. Hydroxyl and N-acetylglucosamine functionalized dendrimers, which are not bound by the galectin-3 CRD, do not cause any change in galectin-3 clustering or cellular aggregation [7,57].

Of the two cell lines reported here, HT-1080 cells produce the least endogenous galectin-3. Thus, excess galectin-3 was not expected to have the dramatic impact on migration trends that was observed. HT-1080 cells likely rely less on galectin-mediated cellular processes for binding, but the results here suggest that galectin-3 can become a major component of the migration process even for cells such as HT-1080 cells that lack significant amounts of endogenous galectin-3. Although lactose-functionalized dendrimers restore more cellular migration for DU 145 cells than for HT-1080 cells (Figure 6), the impact of lactose-functionalized dendrimers on galectin-3-mediated migration was significant even for the HT-1080 cells.

Cellular uptake of galectin-3 in the presence of lactose-functionalized G(6)-PAMAM was somewhat reduced relative to uptake in the absence of the glycodendrimer, as evidenced by the amount of fluorescence in the immunofluorescence assay when antibody was used at 4 °C (when endocytosis of the antibody is inhibited, and therefore the antibody primarily binds to extracellular galectin-3). Comparison between the results when the antibody was used at 37 °C and at 4 °C indicates that about 55% of the galectin-3 was endocytosed in the absence of glycodendrimer compared to about 30% in the absence of galectin-3. This is as expected, since galectin-3/glycodendrimer clusters are significantly larger than individual molecules of galectin-3. We have previously shown that all four of the lactose-functionalized dendrimers used herein are taken up by cancer cells [49].

In addition to changes in cellular uptake, less galectin-3 was detected in the immunofluorescence assays when lactose-functionalized G(6)-PAMAM was present (compare Figure 10A with Figure 10C). This finding supports our proposal that exogenous galectin-3 is diverted from interactions with the cancer cells upon binding by the glycodendrimers, restoring migration in the scratch assay (as shown schematically in Figure 13c,d).

The purpose of the viability assays performed here was to exclude cytotoxicity as the main cause of the observed changes in migration. If no change in cellular migration into the scratch upon application of the glycodendrimers had been observed, then this could have meant that lack of migration was a result of lower viability. Instead, the results upon addition of glycodendrimers with galectin-3 showed a decrease in viability and an increase in migration (relative to addition of galectin-3 alone). Both HT-1080 and DU 145 cells are adherent cell lines that detach from the surface upon cell death. Thus, only live cells are visible in the scratch assay; live cells must be responsible for wound healing in the scratch assay.

Although none of the glycodendrimers have a deleterious effect on cellular viability per se (Figure 11a), a reduction in cellular viability was observed when glycodendrimers were added with galectin-3. The cellular crosslinking that occurred upon addition of galectin-3 to the cells reduces cellular viability (Figure 11b), but the addition of galectin-3 with glycodendrimers, which reduced the cellular crosslinking and restored cellular migration, further reduces cellular viability (Figure 12). When cells cannot migrate (e.g., when galectin-3 is added), their viability is reduced. When galectin-3/carbohydrate interactions at the cell surface are inhibited (e.g., when galectin-3 and glycodendrimers are added), cellular viability is further reduced even while migration is enhanced. We previously reported that glycodendrimers increase apoptosis in cancer cells, partially overcoming the protections afforded by endogenous galectin-3 [49]. The disruption of crosslink-dependent survival when glycodendrimers remove intercellular contacts during the scratch assay could trigger cell death via anoikis, explaining the observed reduction in cellular viability when glycodendrimers are added with galectin-3. These results suggest that cellular mobility and viability both require optimal cell surface interactions that are modulated by galectin-3/carbohydrate interactions at the cell surface.

## 4. Materials and Methods

### 4.1. General Methods

General reagents were purchased from MilliporeSigma (Burlington, MA, USA) and Thermo Scientific (Waltham, MA, USA) Chemical Companies. PAMAM dendrimers were purchased from Dendritech, Inc. (Midland, MI, USA). The CellTiter-Glo Luminescent Cell Viability Assay (CTG) was purchased from Promega (G7572, Madison, WI, USA). Lactose-functionalized PAMAM dendrimers were synthesized according to published procedures [31]. Characterization data is also reported in this manuscript [31]. Full-length galectin-3 (amino acids 1–250) and the galectin-3 CRD (amino acids 112–250) were obtained from BL21(DE3)-pRIL *E. coli* (Stratagene/Agilent Technologies, La Jolla, CA, USA) grown using an autoinduction protocol that lacks IPTG, according to the published protocol [58]. Phosphate-buffered saline (PBS) is 1× diluted from the Cold Spring Harbor 10× PBS protocol (https://cshprotocols.cshlp.org/content/2016/2/pdb.rec079087.full?text_only=true, accessed June 2026).

### 4.2. Cell Culture

Two immortalized cancer cell lines were selected for analysis based on their robustness and galectin-3 expression levels. DU 145 (ATCC (Manassas, VA, USA): HTB-81) prostate carcinomas were cultured in media consisting of Dulbecco’s modified Eagle’s medium (DMEM: Gibco, 61100) with 1.8 g/L NaHCO_3_ and supplemented with MEM vitamin (Gibco, 11126), Penn/strep (Gibco (Chicago, IL, USA), 15140) or Primocin (InvivoGen (San Diego, CA, USA), ant-pm-1), NEAA (Gibco, 11140), EAA (Gibco, 11130) and 10% *v*/*v* fetal bovine serum (FBS). HT-1080 (ATCC: CCL-121) fibrosarcomas were cultured in media consisting of Dulbecco’s modified Eagle’s medium (DMEM: Gibco, 61100) with 1.8 g/L NaHCO_3_ and supplemented with MEM vitamin (Gibco, 11126), Penn/strep (Gibco, 15140), NEAA (Gibco, 11140), EAA (Gibco, 11130) and 5% *v*/*v* fetal bovine serum (FBS). Cells were cultured in a humidified incubator with 5% CO_2_ and 37 °C. Before confluency, cells were subcultured using 0.25% (*w*/*v*) Trypsin-0.53 mM EDTA. Cells were used at passages of ten or below in all experiments.

### 4.3. Scratch Assay Protocol

A 2-well culture insert (ibidi: 80209) was attached to the bottom of each well in a 24-well plate. Complete media (70 μL) containing 100,000 cells was aliquoted to each well of the 2-well insert and briefly shaken. The plate was then placed in the incubator until cells adhered to the plate in a mono-confluent layer, about 8–12 h. The plate was removed from the incubator, and inserts were gently removed using sterile tweezers. Each well was gently washed with about 1 mL of complete media. Galectin-3 was diluted in sterile 1× PBS to 0.5–0.6 mg/mL. The CRD was diluted in PBS to 0.6 mg/mL. The total volume of PBS in each well was 250 μL, and no media was present during the scratch assay. Dendrimers and protein were aliquoted into respective wells to a final concentration of: CRD = 28.3 μM, galectin-3 = 7.4 μM, G(2) = 94.0 μM in dendrimer (1.41 mM in lactose), G(3) = 56.6 μM in dendrimer (1.13 mM in lactose), G(4) = 20.6 μM in dendrimer (930 µM in lactose), G(6) = 10.3 μM in dendrimer (1.03 mM in lactose). Dendrimer concentrations were used such that the concentration of lactose was approximately 1 mM for all experiments, which was based on concentrations used in cellular aggregation assays with the same glycodendrimers [6]. Concentrations of monomeric lactose used for results shown in Figure 8 were 850 µM, 2.54 mM, and 3.39 mM. The control wells had 250 μL of PBS without any additional additives. Plates were then stored in the incubator and removed every 2 or 4 h to take photos of the scratch. Images were collected on an inverted scope with a 4× objective. Photographs were later stitched together using GIMP2 version 2.0 or ImageJversion 1.54g [31], cropped, and the scratch area was quantified using T-scratch. Experiments were reproduced at least three times. Because the size of the scratch inevitably varies across individual trials, causing the amount of open area to vary, controls for galectin-3 alone and PBS alone were performed for every plate, and experiments were normalized to standardize the t = 0 time point.

### 4.4. Immunofluorescence Microscopy Protocol

To replicate the surface that the cells bind to in the scratch assay, slides were cut from a TC 24-well plate and then placed on the bottom of the wells of another 24-well plate. To this slide, an insert was attached, and cells were added in the same method as the scratch assay. After the assay had run for either 4 or 12 h, the wells were rinsed with whole media and filled with 250 μL of 1:50 MAb (Alexa Fluor 488-conjugated galectin-3 antibody, BioLegend (San Diego, CA, USA)) in whole media and incubated for 1 h at 37 °C or 4 °C. While protecting the slide from light, the antibody solution was removed, the well was covered with 4% paraformaldehyde for 15 min, and then the well was covered with PBS-T for 3 min. When the PBS-T was removed, the slides were mounted to a No 1 glass cover slip with Prolong Gold and allowed to cure overnight at RT in the dark. When cured, the edges were sealed with clear nail polish, and the samples were stored at 4 °C until they were visualized by fluorescent microscopy on a Zeiss 510 inverted confocal microscope. Fluorescence analysis was performed using ImageJ (NIH). The fluorescence from three background areas was averaged and subtracted from the average of three regions of interest for each image to obtain corrected fluorescence intensity values. Values summarized in Figure 10 are provided in Tables S12 and S13 in the Supplementary Information.

### 4.5. Cellular Viability Assay Protocol

Complete media (90 μL) containing 3000 cells was aliquoted into each well of a 96-well plate. The plate was then placed in the CO_2_ incubator for 18–24 h. On day two of the assay, 30 μL of PBS solution containing additives was added to each well. Cells were allowed to incubate for 72 h. The plate was removed from the incubator and let sit for 20 min before adding 20 μL of CTG solution into each well in the dark. Using a 96-well plate reader, the plate was shaken for two minutes, allowed to sit for 10 min, and then read for luminescence signal.

### 4.6. Statistical Analysis

Statistical analysis was performed using the ordinary one-way ANOVA in GraphPad Prism, version 10.6.1 and is provided in Tables S14–S21 in the Supplementary Information. * *p* < 0.05, ** *p* < 0.01, *** *p* < 0.0001, **** *p* < 0.0001, ns = not significant (*p* > 0.05). Data in graphs are shown as mean with standard deviation.

## 5. Conclusions

Addition of galectin-3 inhibits cellular migration and reduces cellular viability by crosslinking cells. Studies reported herein indicate that addition of lactose-functionalized PAMAM dendrimers concurrently with addition of galectin-3 restores cellular migration by providing a competitive alternative for galectin-3 binding to cell surface carbohydrates. The reduction in interactions between carbohydrates on the cancer cell surface and galectin-3 that serve to crosslink the cells and prevent the cells from migrating is reduced in the presence of the lactose-functionalized dendrimers. Removal of the cell surface crosslinking interactions, however, causes a reduction in cellular viability when lactose-functionalized dendrimers are added in conjunction with exogenous galectin-3. The reduction in cellular viability upon addition of galectin-3 presumably occurs because cells are tethered together too tightly, but when too many cellular crosslinks are eliminated (e.g., when lactose-functionalized dendrimers and galectin-3 are added in concert), then cellular viability is further reduced. These results indicate that cell surface galectin-3/carbohydrate interactions play an important role in the modulation of cellular migration. The cell surface galectin-3/carbohydrate interactions are in addition to the intracellular galectin-3/protein interactions that have been previously reported to modulate cellular migration. Galectin-3-mediated migration almost certainly contributes to cancer cell invasion and metastasis, particularly in cancers with high expression levels of galectin-3. Therefore, elucidating the roles of both extracellular and intracellular galectin-3/ligand interactions in cellular migration is of utmost importance.

## Data Availability

The original contributions presented in this study are included in the article/Supplementary Materials. Further inquiries can be directed to the corresponding authors.

## Supplementary Materials

The following supporting information can be downloaded at: https://www.mdpi.com/article/10.3390/ijms27167145/s1.

## Abbreviations

The following abbreviations are used in this manuscript:

BM-MSCs	bone marrow mesenchymal stem cells
CRD	carbohydrate recognition domain
CTG	CellTiter-Glo luminescent cell viability assay (Promega)
G(2)	lactose-functionalized second-generation poly(amidoamine) dendrimer
G(3)	lactose-functionalized third-generation poly(amidoamine) dendrimer
G(4)	lactose-functionalized fourth-generation poly(amidoamine) dendrimer
G(6)	lactose-functionalized sixth-generation poly(amidoamine) dendrimer
IC_50_	half maximal inhibitory concentration
IPTG	isopropyl thiogalactoside
MMP	matrix metalloproteinase
NTD	N-terminal domain
PAMAM	poly(amidoamine) dendrimer
PBS	phosphate-buffered saline
PSVII	paris saponin VII
ROMP	ring-opening metathesis polymerization
SDS	sodium dodecyl sulfate
TNBC	Triple-negative breast cancer
