# Supplementary material for: Extracellular Galectin-3/Carbohydrate Interactions Modulate Cancer Cellular Migration
Source: Int J Mol Sci. 2026 Aug 9;27(16):7145. doi: 10.3390/ijms27167145 (PMC13512990; doi:10.3390/ijms27167145)
Supplement: Supplementary file 1 [file ijms-27-07145-s001.zip › ijms-4438210-supplementary.pdf]

Extracellular Galectin-3/Carbohydrate Interactions Modulate Cancer Cellular Migration

Mackenzie S. Fricke, Ramat S. Tahir, Hazal K. Ural and Mary J. Cloninger *

Department of Chemistry and Biochemistry, Montana State University, Bozeman, MT 59717 USA; mackenzie.fricke.24@gmail.com (M.S.F.); ramattahir@montana.edu (R.S.T.); hkaterinau@gmail.com (H.K.U.)

* Correspondence: mcloninger@montana.edu

Supplementary Information

Table of Contents **Page**

Figure S1. Micrographs of DU 145 migration into the scratch with exogenous galectin-3 1
Figure S2. Micrographs of DU 145 migration into the scratch with PBS 1
Figures S3-S6. Micrographs of DU 145 migration into the scratch with lactose-functionalized PAMAM dendrimers and galectin-3. 2-3
Figure S7. Plots for DU 145 migration with lactose-functionalized PAMAMs 4
Figures S8-S11. Micrographs of DU 145 migration with lactose-functionalized PAMAMs 4-6
Figures S12. Micrographs of HT-1080 migration with galectin-3 7
Figure S13. Micrographs of HT-1080 migration with PBS 7
Figures S14-S17. Micrographs of HT-1080 migration with lactose-functionalized PAMAMs and galectin-3 7-8
Figure S18. Plots for HT-1080 migration with lactose-functionalized PAMAMs 9
Figures S19-S22. Micrographs of HT-1080 migration with lactose functionalized PAMAMs 10-11
Figure S23. Micrographs of DU 145 with the galectin-3 CRD 12
Figure S24. Micrographs of DU 145 with the CRD and lactose-functionalized G(6)-PAMAM 13
Figures S25-S27. Micrographs of DU 145 with galectin-3 and lactose 13-14
Table S1. Cellular viability values for DU 145 cells with the galectin-3 CRD 15
Tables S2-S5. Cellular viability values for DU 145 cells with lactose-functionalized PAMAMs 16-19
Tables S6-S9. Cellular viability values for DU 145 cells with galectin-3 and lactose-functionalized PAMAMs 19-20
Table S10. Cellular viability values for DU 145 cells with galectin-3 20
Table S11. IC_{50} summary of results for DU 145 cells with galectin-3 21
Tables S12-S13. Fluorescence intensity values for data shown in Figures 9 and 10 22-24
Tables S14-S21. P values for data shown in Figures 4, 5, 7-10, S2, and S18 24-26

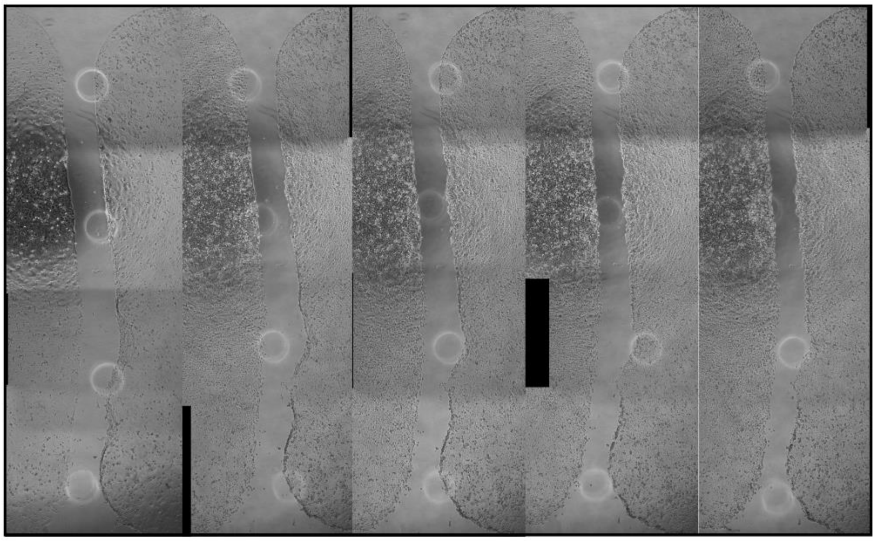

Figure S1. DU 145 migration into the scratch over 16 hours with exogenously added galectin-3

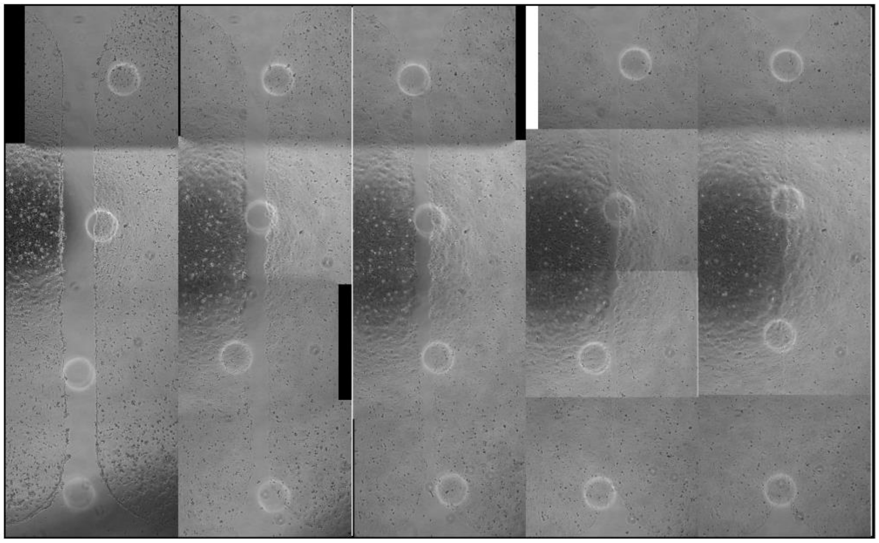

Figure S2. DU 145 migration into the scratch over 16 hours with PBS.

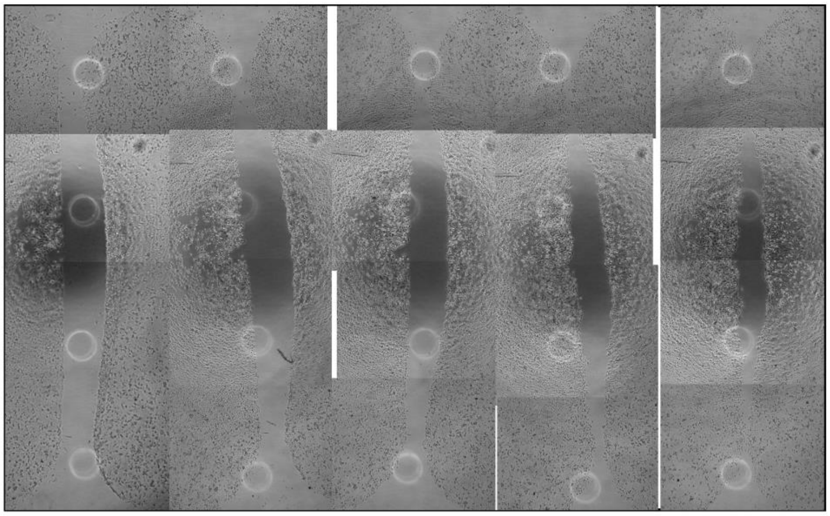

Time (hours): 0 4 8 12 16

Figure S3. DU 145 migration into the scratch with exogenous galectin-3 and lactose-functionalized G(2)-PAMAM.

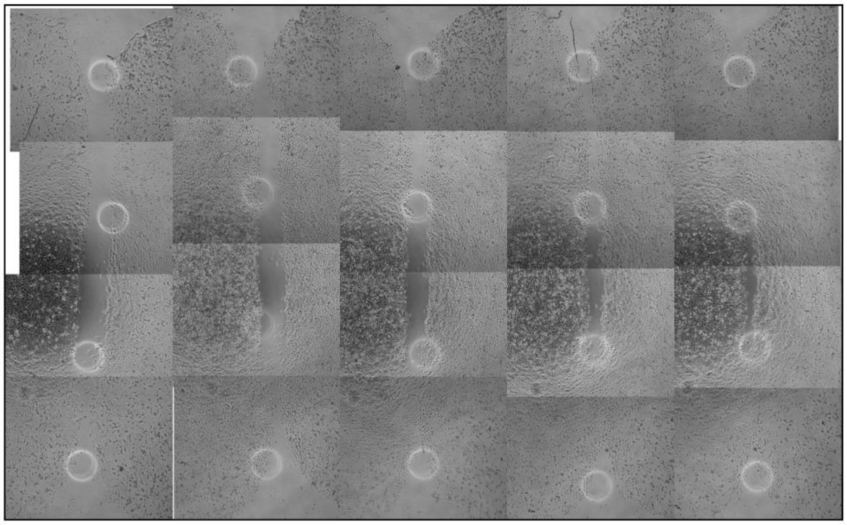

Time (hours): 0 4 8 12 16

Figure S4. DU 145 migration into the scratch with galectin-3 and lactose-functionalized G(3)-PAMAM.

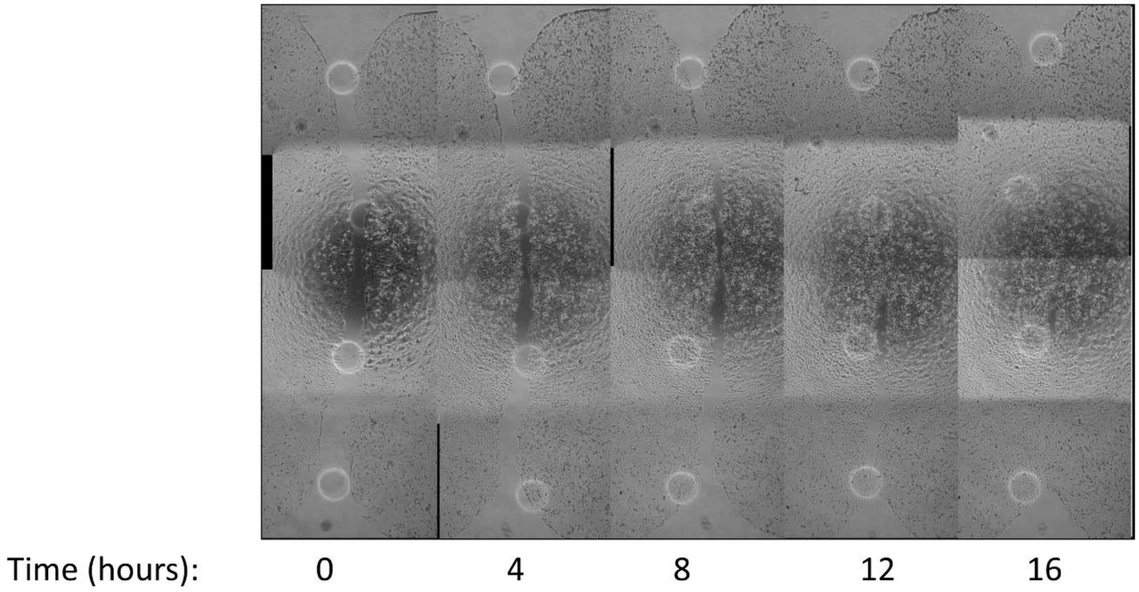

Figure S5. DU 145 migration into the scratch with galectin-3 and lactose-functionalized G(4)-PAMAM.

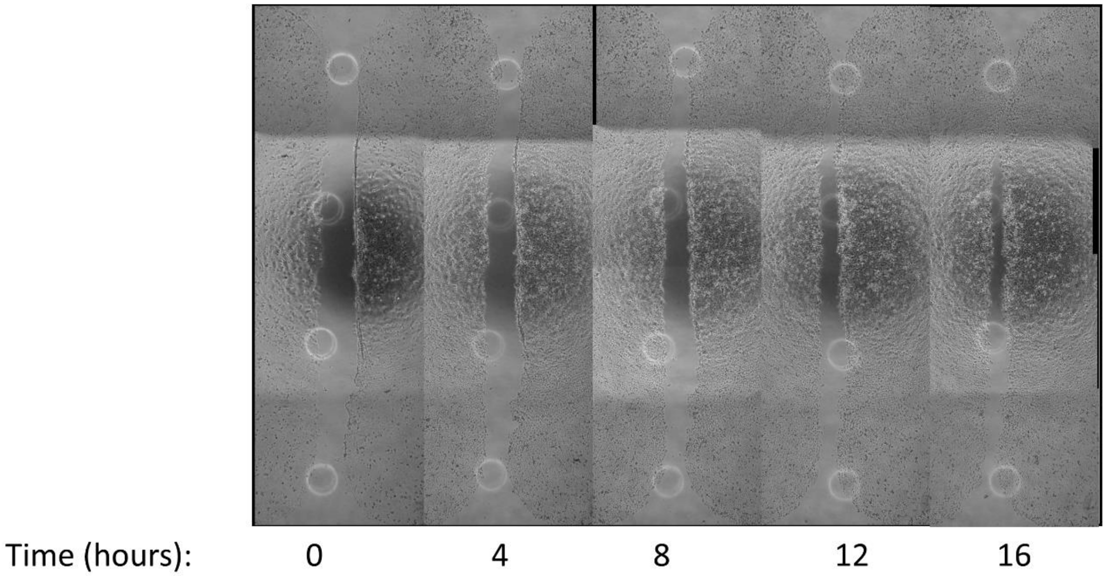

Figure S6. DU 145 migration into the scratch with galectin-3 and lactose-functionalized G(6)-PAMAM.

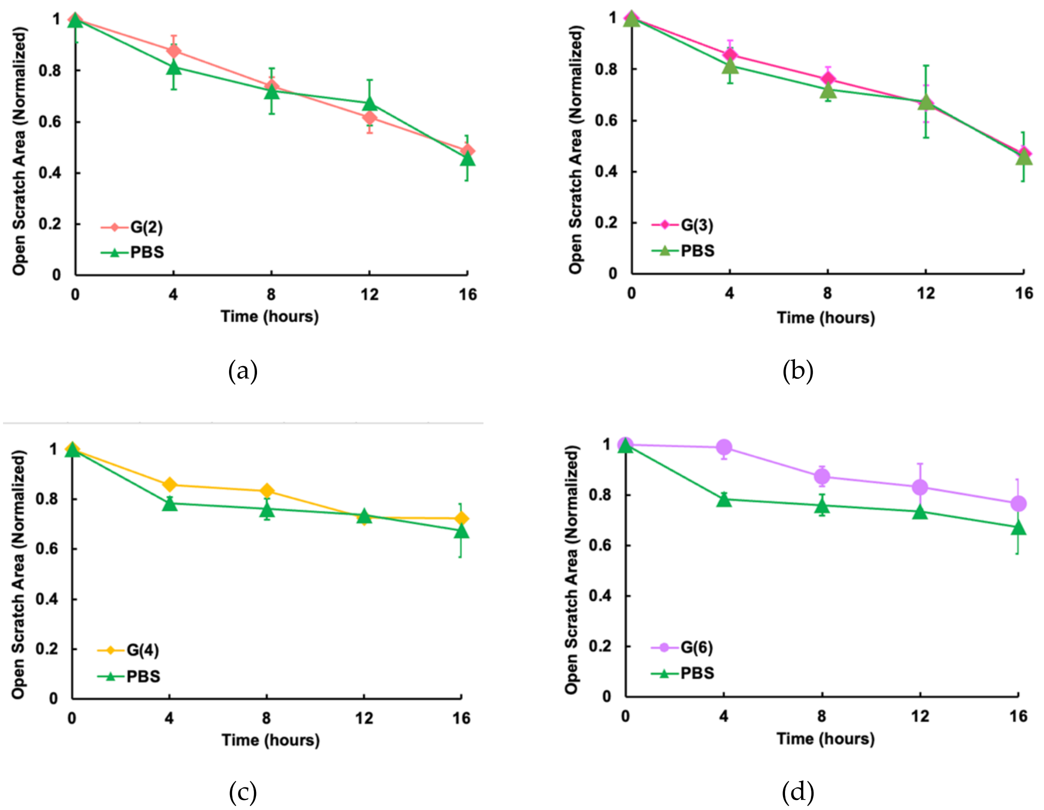

DU-145

Figure S7. DU 145 migration with lactose functionalized glycodendrimers but not galectin-3

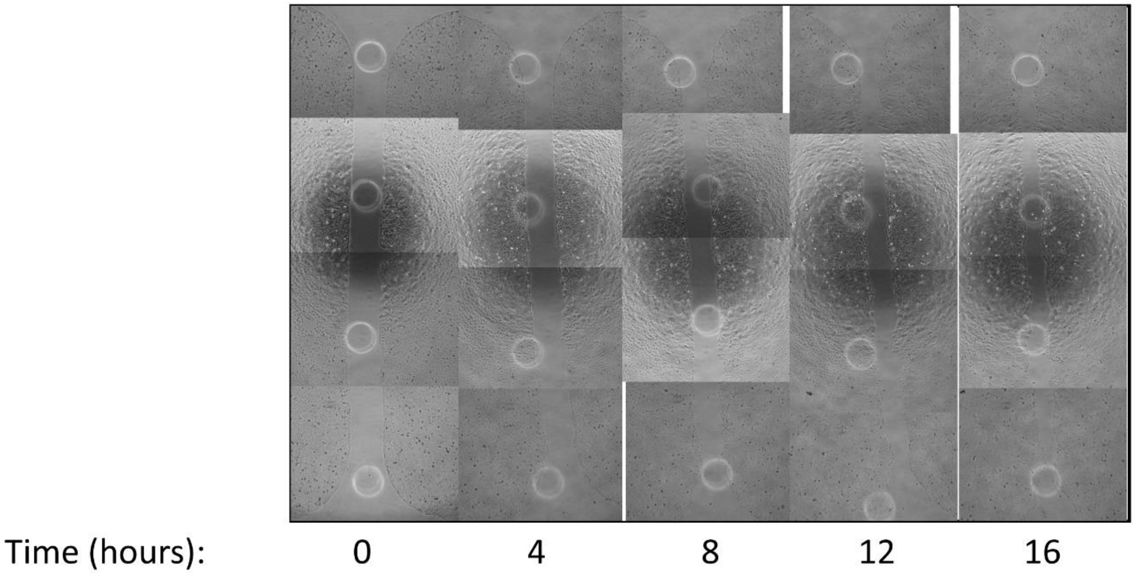

Figure S8. DU 145 migration into the scratch with G(2)-PAMAM.

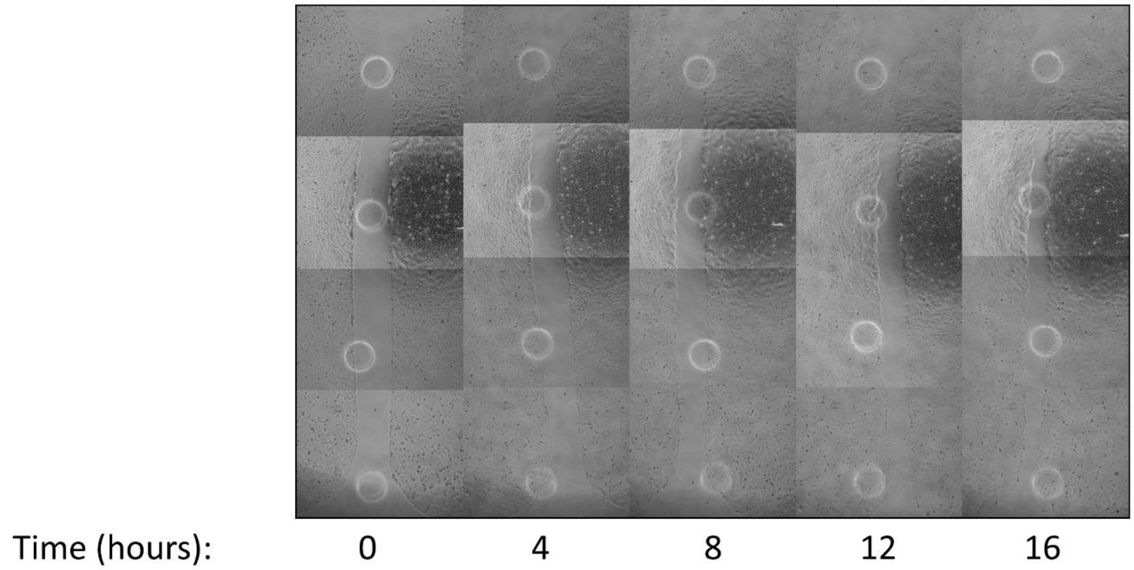

Figure S9. DU 145 migration into the scratch with lactose-functionalized G(3)-PAMAM.

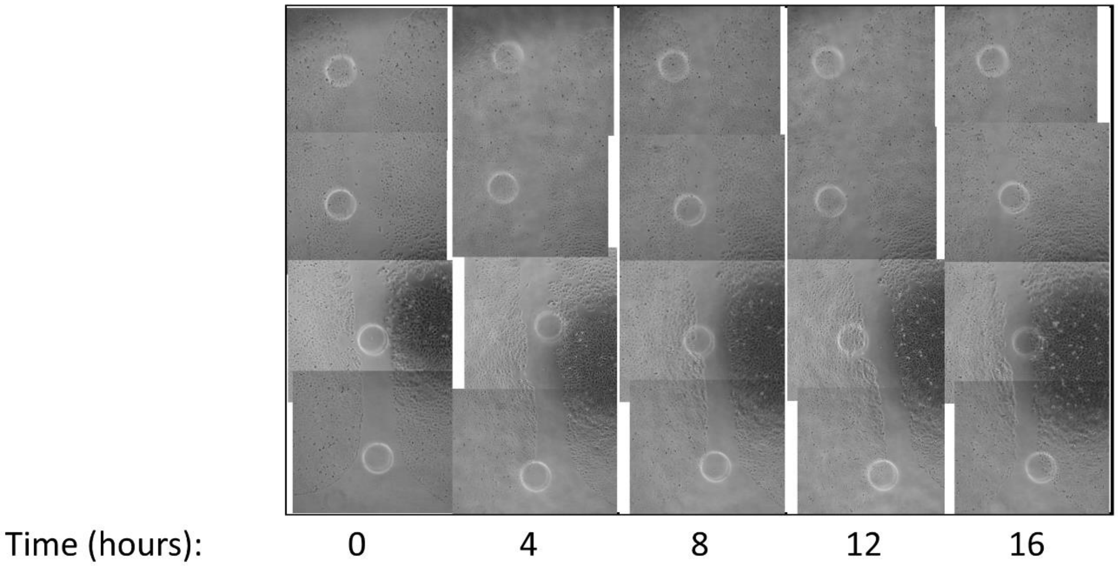

Figure S10. DU 145 migration with lactose-functionalized G(4)-PAMAM.

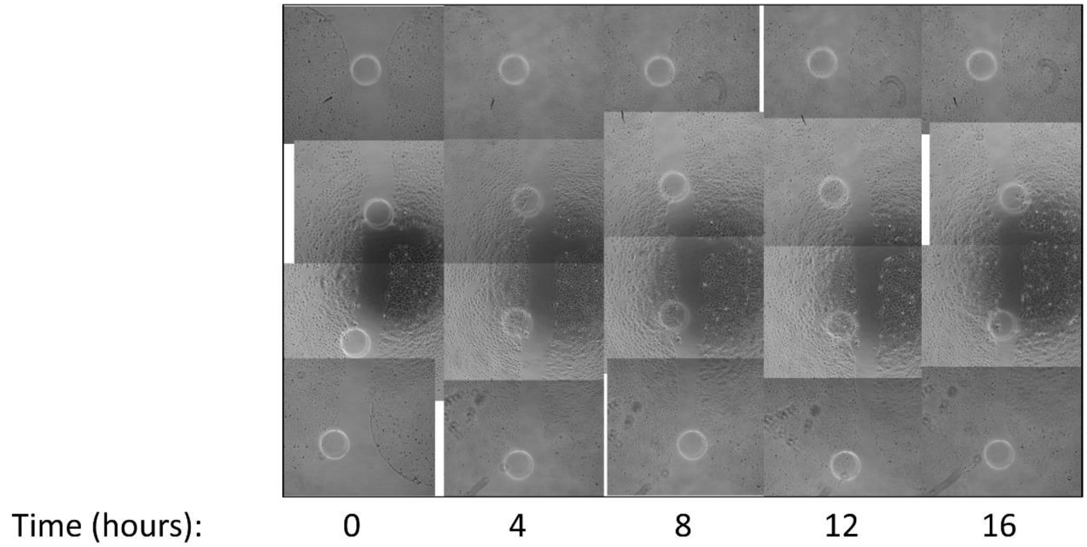

Figure S11. DU 145 migration with lactose-functionalized G(6)-PAMAM.

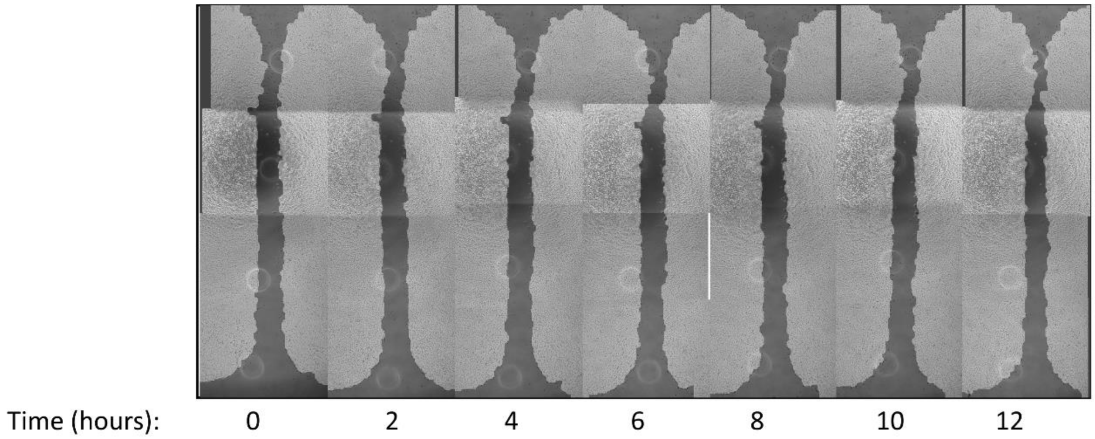

Figure S12. HT-1080 migration into the scratch with galectin-3 over 12 hours.

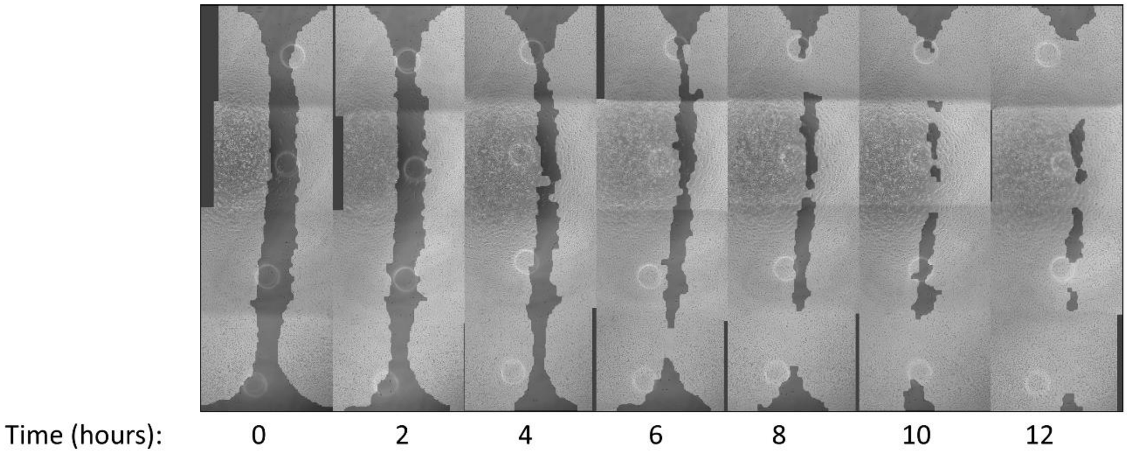

Figure S13. HT-1080 migration into the scratch with PBS.

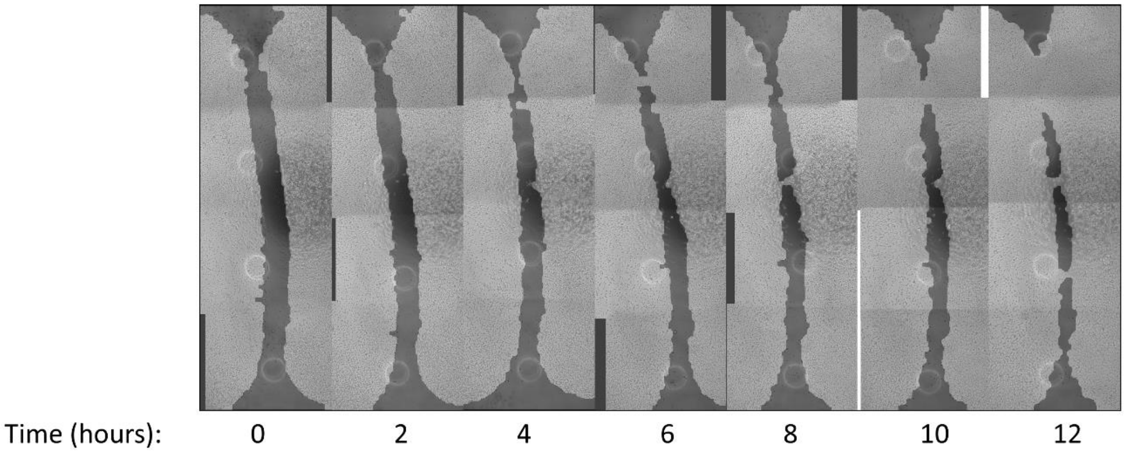

Figure S14. HT-1080 migration into the scratch with galectin-3 and lactose-functionalized G(2)-PAMAM.

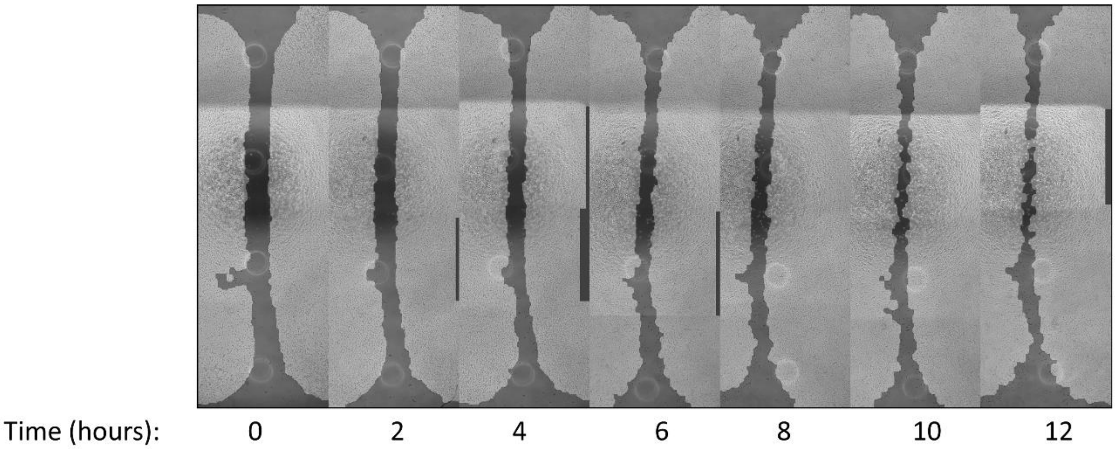

Figure S15. HT1080 migration into the scratch with galectin-3 and lactose-functionalized G(3)-PAMAM.

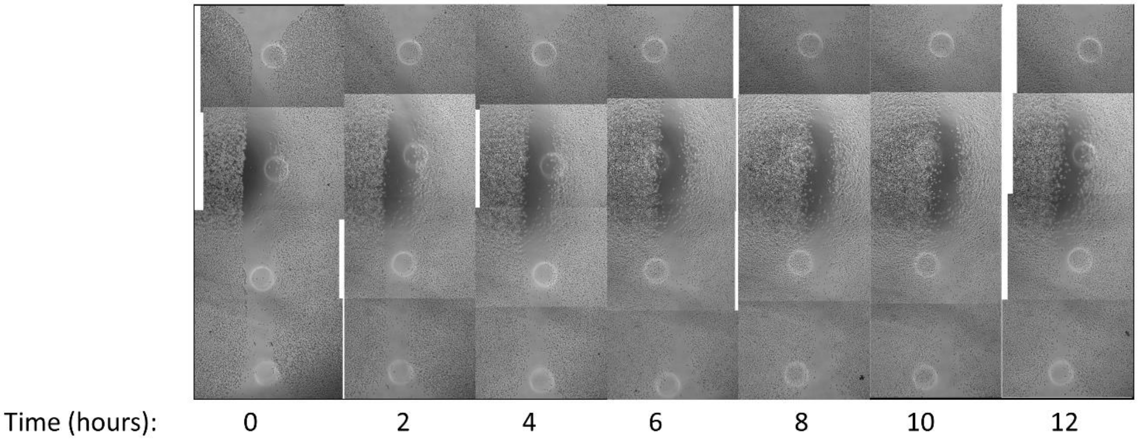

Figure S16. HT-1080 migration into the scratch with galectin-3 and lactose-functionalized G(4)-PAMAM.

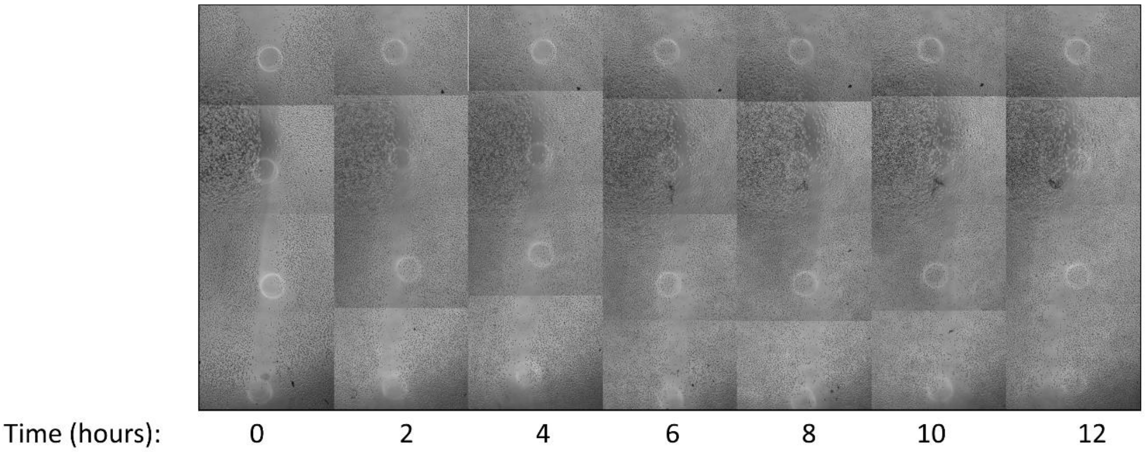

Figure S17. HT-1080 migration into the scratch with galectin-3 and lactose-functionalized G(6)-PAMAM.

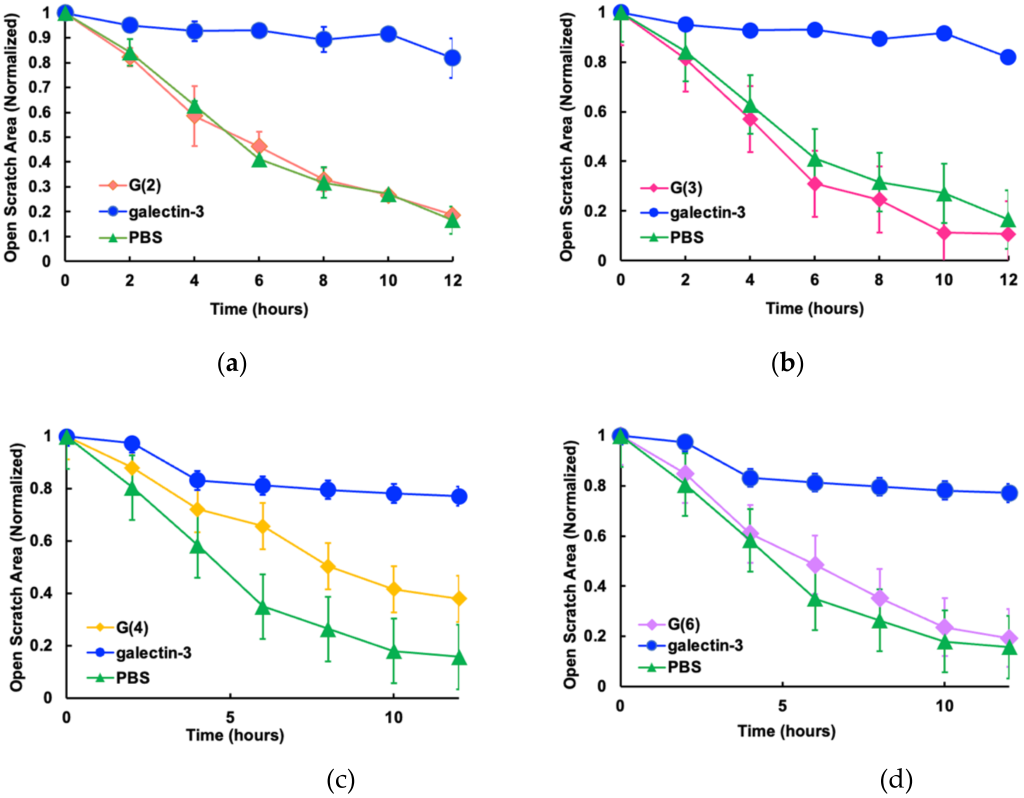

Figure S18. HT-1080 migration with glycodendrimers but not galectin-3

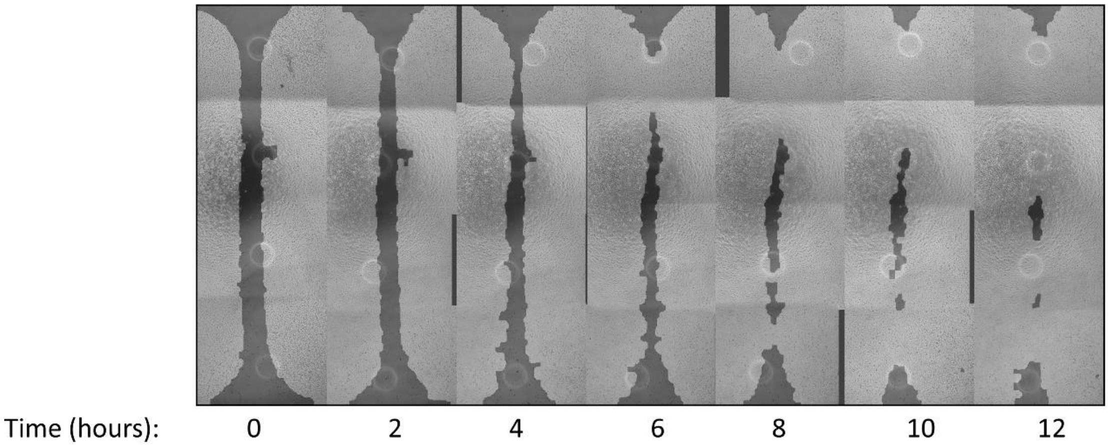

Figure S19. HT-1080 migration into the scratch with lactose-functionalized G(2)-PAMAM.

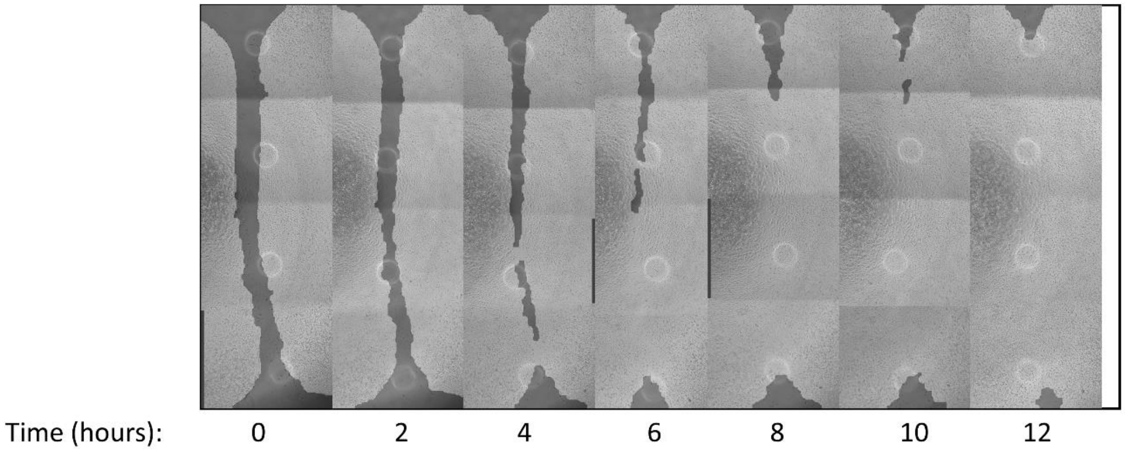

Figure S20. HT-1080 migration into the scratch with lactose-functionalized G(3)-PAMAM.

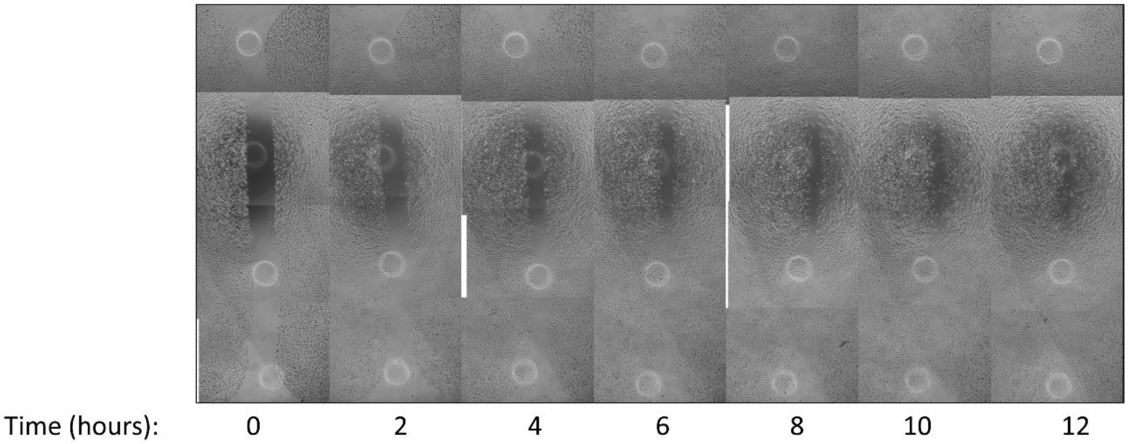

Figure S21. HT-1080 migration into the scratch with lactose-functionalized G(4)-PAMAM.

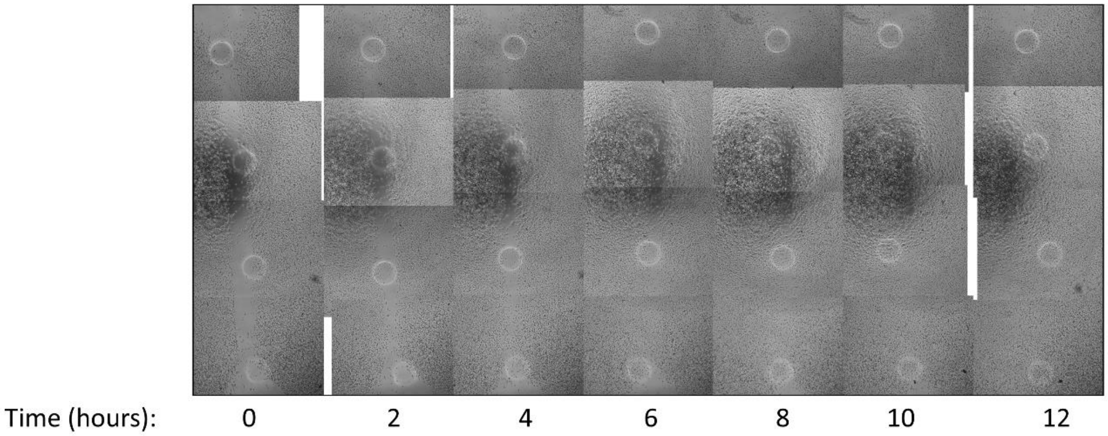

Figure S22. HT-1080 migration into the scratch with lactose-functionalized G(6)-PAMAM.

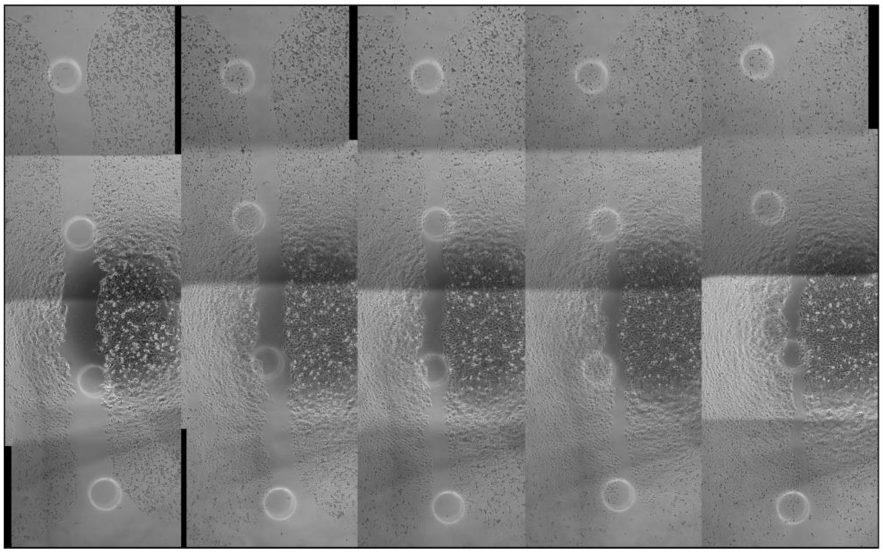

Time (hours): 0 4 8 12 16

Figure S23. DU 145 migration into the scratch with the galectin-3 CRD.

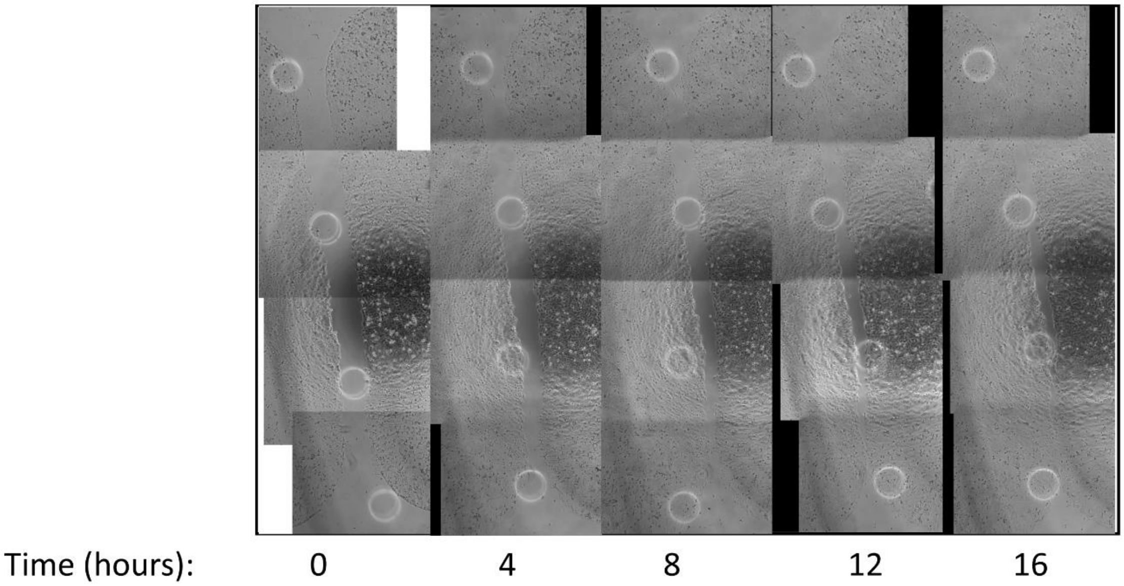

Figure S24. DU 145 migration into the scratch with the CRD and lactose-functionalized G(6)-PAMAM.

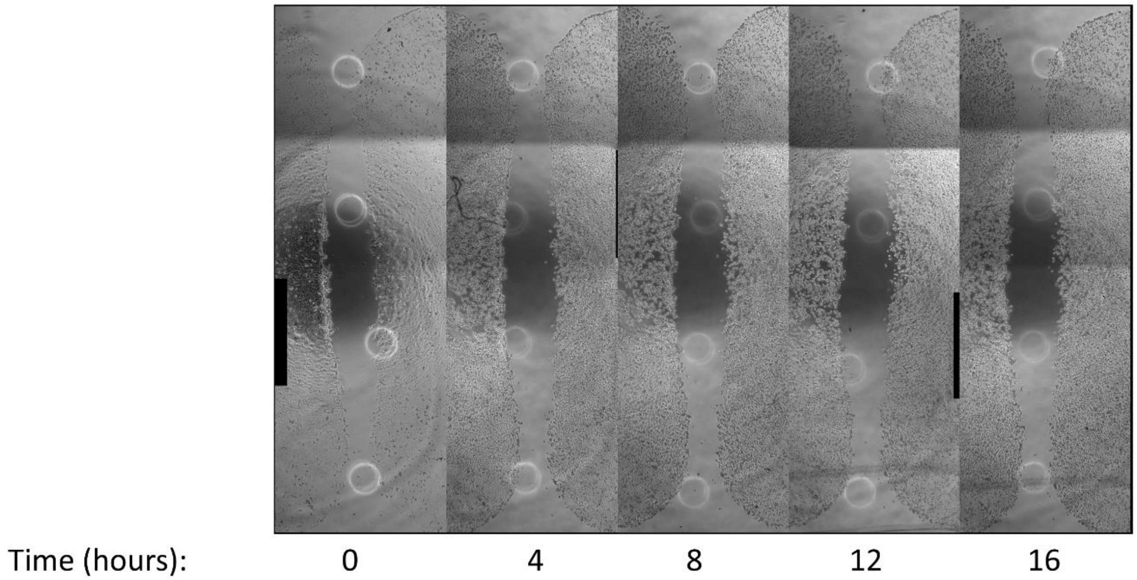

Figure S25. DU 145 migration into the scratch with galectin-3 and 1X monomeric lactose (0.848 mM).

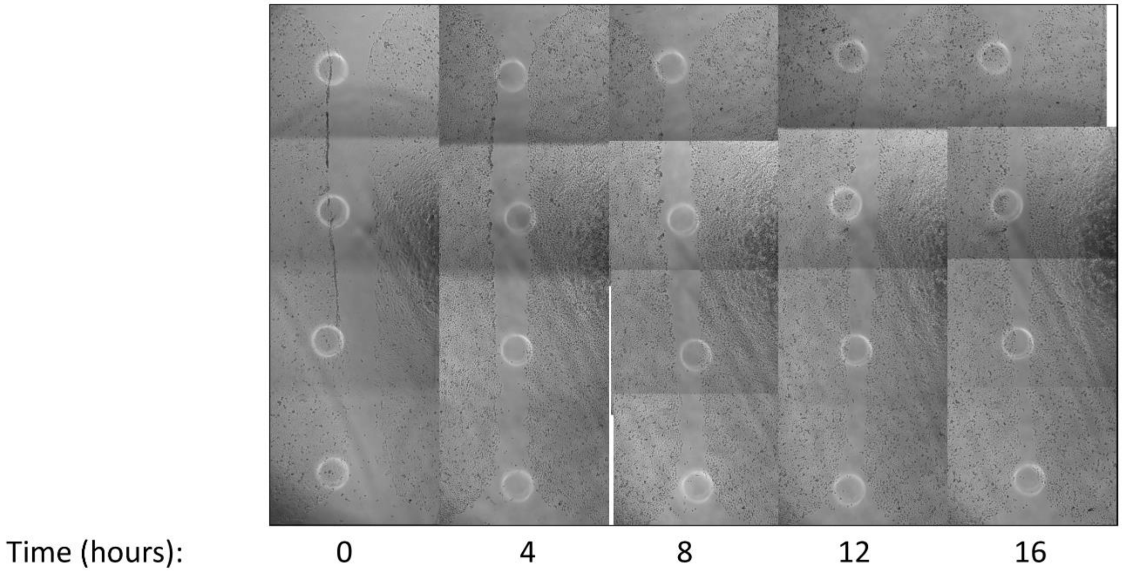

Figure S26. DU 145 migration into the scratch with galectin-3 and 3X monomeric lactose (2.54 mM).

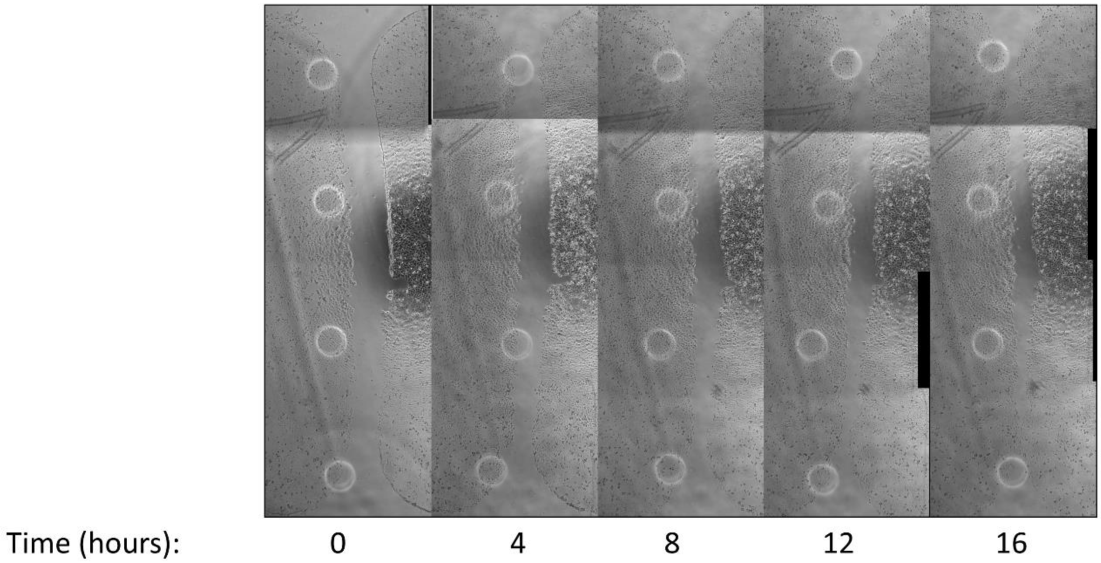

Figure S27. DU 145 migration into the scratch with galectin-3 and 4X monomeric lactose (3.39 mM).

Table S1. Cellular viability results with CRD for DU 145 cells.

[CRD], μM	Trial 1 (normalized)	Trial 1 (lumens)	Trial 2 (normalized)	Trial 2 (lumens)
0	1.000	46572	1.000	40649
5	1.041	47863	1.063	42369
7	1.120	50316	1.039	41700
9	1.149	51207	0.988	40316
11	1.234	53845	0.874	37219
13	1.233	53833	0.955	39433
15	1.217	53314	0.915	38335
17	0.853	42001	0.812	35543
19	1.106	49858	0.764	34245
29	0.922	44146		
0.5% SDS	0.000	15458	0.000	13476
[CRD],5-29 μM	Average	1.017		
	Standard Deviation	0.144		

Table S2. Cellular viability results with lactose functionalized G(2)-PAMAM dendrimers for DU 145 cells.

[G(2)], μM	Trial 1 (normalized)	Trial 1 (lumens)	Trial 2 (normalized)	Trial 2 (lumens)	Trial 3 (normalized)	Trial 3 (lumens)	Trial 4 (normalized)	Trial 4 (lumens)
0	1.000	37632	1.000	37387	1.000	1573	1.000	836128
10	1.210	41215						
20	1.375	44051						
30	1.304	42825						
40	1.181	40733						
50	1.012	37836						
60	1.011	37816						
70	1.201	41074						
70			0.886	34612			0.892	757806
75			0.948	36110				
80	1.108	39472	0.890	34700			0.891	756603
85			0.884	34551				
90	1.090	39176	0.923	35504			0.826	709725
95			1.125	40441				
100	0.923	36320	1.157	41205	0.934	1504		
105			1.121	40337				
110	1.002	37665	0.933	35758	1.421	2015		
120					1.024	1598		
0.5% SDS	0.000	20538	0.000	12989	0.000	525	0.000	108462
[G(2)], 10-120 μM	Average	1.049						
	Standard Deviation	0.162						

Table S3. Cellular viability results with lactose functionalized G(3)-PAMAM dendrimers for DU 145 cells

[G(3)], μM	Trial 1 (normalized)	Trial 1 (lumens)	Trial 2 (normalized)	Trial 2 (lumens)	Trial 3 (normalized)	Trial 3 (lumens)	Trial 4 (normalized)	Trial 4 (lumens)	Trial 5 (normalized)	Trial 5 (lumens)
0	1	37632	1	38495	1	5478	1	4121	1	4401
10	1.117933818	39648	1.000724	38512.33	0.688	3790				
20	1.294210557	42661.333	1.016222	38883.5	0.749	4124				
30	1.287093189	42539.667	0.908306	36299			0.875	3616		
40	1.402433555	44511.333	0.923087	36653			0.790	3271		
50	1.089093852	39155	0.878671	35589.25					0.918	4052
60	0.996470565	37571.667	1.152834	42155.25					0.935	4124
70	0.989333697	37449.667	1.121016	41393.25						
80	1.08431644	39073.333	1.033404	39295						
90	1.032037907	38179.667	1.058248	39890						
100	0.795253788	34132								
110	0.85892011	35220.333								
120	0.856268159	35175								
0.5% SDS	0	20537.667	0	14545.75	0	75	0	75	0	138
[G(3)], 10-120 μM	Average	0.9944692								
	Standard Deviation	0.1661893								

Table S4. Cellular viability results with lactose functionalized G(4)-PAMAM dendrimers for DU 145 cells

[G(4)], μM	Trial 1 (normalized)	Trial 1 (lumens)	Trial 2 (normalized)	Trial 2 (lumens)	Trial 3 (normalized)	Trial 3 (lumens)
0	1	43218.5	1	37838.75	1	836127.5
5			0.953357	36680.5	1.110397	916459.7
8	0.910904226	41170.5				
10	0.845213495	39660.5	1.029226	38564.5	1.004065	839085.7
12	1.405324865	52535.5				
14	1.142322668	46490				
15			1.033837	38679	0.909915	770575.7
16	1.079176908	45038.5				
18	1.137015205	46368				
20	1.153742414	46752.5	0.979049	37318.5		
22	1.014639027	43555				
24	0.885084724	40577				
25			1.060425	39339.25		
26	0.973788963	42616				
30	0.87129402	40260	1.197556	42744.5		
35			1.051123	39108.25		
40			1.047851	39027		
45			0.979351	37326		
0.5% SDS	0	20232	0	13006.5	0	108461.7
[G(4)], 5-45 μM	Average	1.0336809				
	Standard Deviation	0.1220554				

Table S5. Cellular viability results with lactose functionalized G(6)-PAMAM dendrimers for DU 145 cells

[G(6)], μM	Trial 1 (normalized)	Trial 1 (lumens)	Trial 2 (normalized)	Trial 2 (lumens)	Trial 3 (normalized)	Trial 3 (lumens)
0	1	39797	1	44313	1	1573
2	1.043	40896	0.803	40498		
4	1.232	45759	0.940	43149		
6	1.151	43675	0.880	41985		
8	0.860	36203	0.962	43570	0.753	1314
10	0.907	37414	0.637	37270	1.409	2002
12	0.881	36745			0.811	1375
14	0.776	34034				
16	0.679	31535				
18	0.611	29794				
0.5% SDS	0	14067	0	24911	0	525
[G(6)], 2-18 μM	Average	0.9020587				
	Standard Deviation	0.2051635				

Table S6. Cellular viability results with lactose functionalized G(2)-PAMAM dendrimers and 5 μM galectin-3 for DU 145 cells

5 μM Galectin-3						
[G(2)], μM	Trial 1 (normalized)	Trial 1 (lumens)	Trial 2 (normalized)	Trial 2 (lumens)	Average (normalized)	Standard Deviation (normalized)
0	1	24071	1	772158		
90	0.737	21475	0.625	555645	0.681	0.0559
0.5% SDS	0	14200	0	194665		

Table S7. Cellular viability results with lactose functionalized G(3)-PAMAM dendrimers and 5 μM galectin-3 for DU 145 cells

5 μM Galectin-3						
[G(3)], μM	Trial 1 (normalized)	Trial 1 (lumens)	Trial 2 (normalized)	Trial 2 (lumens)	Average (normalized)	Standard Deviation (normalized)
0	1	24071	1	780552		
60	0.621	20689	0.615	507772	0.618	0.0030
0.5% SDS	0	15156	0	72541		

Table S8. Cellular viability results with lactose functionalized G(4)-PAMAM dendrimers and 5 μM galectin-3 for DU 145 cells

5 μM Galectin-3						
[G(4)], μM	Trial 1 (normalized)	Trial 1 (lumens)	Trial 2 (normalized)	Trial 2 (lumens)	Average (normalized)	Standard Deviation (normalized)
0	1	780552	1	12612		
20	0.664	542392	0.611	7992	0.637	0.0261
0.5% SDS	0	72541	0	724		

Table S9. Cellular viability results with lactose functionalized G(6)-PAMAM dendrimers and 5 μM galectin-3 for DU 145 cells

5 μM Galectin-3						
[G(6)], μM	Trial 1 (normalized)	Trial 1 (lumens)	Trial 2 (normalized)	Trial 2 (lumens)	Average (normalized)	Standard Deviation (normalized)
0	1	24071	1	1343		
10	0.325	17162	0.248	792	0.286	0.0383
0.5% SDS	0	13842	0	610		

Table S10. Cellular viability results with galectin-3 for DU 145 cells

[galectin-3], μM	Trial 1 (normalized)	Trial 1 (lumens)	Trial 2 (normalized)	Trial 2 (lumens)	Trial 3 (normalized)	Trial 3 (lumens)
0	1	45188	1	38030	1	14370
2			0.956	36947		
4			0.858	34532		
5	0.682	35320			0.679	10312
6			0.641	29177		
7	0.520	30294				
8			0.426	23859		
9	0.226	21190				
10			0.185	17899		
11	0.172	19505				
0.5% SDS	0	14173	0	13341	0	2822

Table S11. IC_{50} summary of results for galectin-3 for DU 145 cells

GraphPad Prism 10		
Absolute IC_{50}, X is concentration		
Best-fit values		
Top	= 1.000	
Baseline	= 0.000	
Bottom		-0.2936
Absolute IC_{50}		6.938
Hill Slope		-2.509
Log Absolute IC_{50}		0.8412
95% CI (profile likelihood)		
Bottom	-4.309 to 0.06501	
Absolute IC_{50}	6.564 to 7.329	
Hill Slope	-3.625 to -1.564	
Log Absolute IC_{50}	0.8172 to 0.8650	
Goodness of Fit		
Degrees of Freedom		10
R squared		0.9884
Sum of Squares		0.01391
Sy.x		0.0373
Constraints		
Top	Top = 1	
Baseline	Baseline = 0	

Table S12. Fluorescence intensity values calculated using ImageJ for images in Figure 9.

Figure 9A	Galectin-3, 4 h, 37 ºC incubation of antibody				Ratio to 9A
	Area	Mean	Integrated density	Corrected total fluorescence	
Region of interest 1	12100	43	524755		
Region of interest 2	22729	47	1067013		
Region of interest 3	22240	48	1069148		
Average for ROIs	19023	46	886972	642811.80	1
Background 1	462	14	6377		
Background 2	1120	10	11268		
Background 3	702	15	10278		
Average background	761.33	13	9308		
Figure 9B	Galectin-3, 12 h, 37 ºC incubation of antibody				
	Area	Mean	Integrated density	Corrected total fluorescence	
Region of interest 1	33507	43	1436786		
Region of interest 2	20043	40	811429		
Region of interest 3	14175	54	767318		
Average for ROIs	22575	46	1005178	657161.47	1.02
Background 1	1560	16	25534		
Background 2	780	17	13318		
Background 3	464	13	5942		
Average background	934.67	15	14931		

*Corrected total fluorescence = Integrated density - Ave area ROI*Ave mean background)

Table S13. Fluorescence intensity values calculated using ImageJ for images in Figure 10.

Figure 10A	Galectin-3 + G(6), 4 h, 37 °C incubation of antibody				
	Area	Mean	Integrated density	Corrected total fluorescence*	Ratio to 10C
Region of interest 1	9384	25.62	240415		
Region of interest 2	22752	33.925	771872		
Region of interest 3	23940	23.053	551891		
Average for ROIs	18692	27.533	521392.67	364897	0.61836392
					62%
Background 1	1890	8.987	16985	38% less galectin-3 is attached	
Background 2	2280	6.884	15696	to the scratch when G(6) is	
Background 3	2244	9.246	20747	present.	
Average background	2138	8.372	17809.33		
Figure 10B	Galectin-3 + G(6), 4 h 4 °C incubation of antibody				
	Area	Mean	Integrated density	CTF	
Region of interest 1	28224	21.599	609623		
Region of interest 2	27132	19.621	532365		
Region of interest 3	37752	16.77	633096		
Average for ROIs	31036	19.33	591694.67	261244	0.4427109
					44%
Background 1	972	10.309	10020		1 - 10B/10A =
Background 2	1748	11.649	20363		0.28406092
Background 3	910	9.984	9085		28% is
Average background	1210	10.647	13156		endocytosed
Figure 10C	Galectin-3, 4 h, 37 °C incubation of antibody				
	Area	Mean	Integrated density	CTF	
Region of interest 1	19630	40.851	801913		
Region of interest 2	32136	29.443	946181		
Region of interest 3	33672	32.468	1093259		
Average for ROIs	28479.33	34.254	947117.67	590101	1
					100%
Background 1	729	15.881	11577		
Background 2	696	14.552	10128		
Background 3	1682	7.175	12068		
Average background	1035.67	12.536	11257.67		

Table S13 Continued

Figure 10D	Galectin-3, 4 h, 4 ºC incubation of antibody				
	Area	Mean	Integrated density	CTF	
Region of interest 1	19662	28.683	563958		
Region of interest 2	21027	25.578	537822		
Region of interest 3	22048	20.337	448399		
Average for ROIs	20912.33	24.866	516726.33	260662	0.44172421
					44%
Background 1	960	12.293	11801		56% is endocytosed
Background 2	768	11.996	9213		
Background 3	874	12.445	10877		
Average background	867.33	12.2446667	10630.33		

Table S14. P values for data shown in Figures 4 and S2 for DU 145 cells. *P≤0.05, **P≤0.01, ***P≤0.0001, ****P≤0.0001, ns = not significant (P>0.05).

	4 h		8 h		12 h		16 h	
	P value	Summary	P value	Summary	P value	Summary	P value	Summary
PBS vs. G(2)	0.3038	ns	0.6037	ns	0.565	ns	0.6589	ns
PBS vs. G(3)	0.4611	ns	0.3349	ns	0.929	ns	0.8632	ns
PBS vs. G(4)	0.2899	ns	0.0815	ns	0.814	ns	0.5709	ns
PBS vs. G(6)	0.0064	**	0.0282	*	0.211	ns	0.3207	ns

Table S15. P values for data shown in Figure 4 for DU 145 cells. *P≤0.05, **P≤0.01, ***P≤0.0001, ****P≤0.0001, ns = not significant (P>0.05).

	4 h		8 h		12 h		16 h	
	P value	Summary	P value	Summary	P value	Summary	P value	Summary
PBS vs. G(2) + galectin-3	0.0144	*	0.056	ns	0.062	ns	0.4625	ns
PBS vs. G(3) + galectin-3	0.0166	*	0.0188	*	0.133	ns	0.0908	ns
PBS vs. G(4) + galectin-3	0.0507	ns	0.0209	*	0.062	ns	0.0281	*
PBS vs. G(6) + galectin-3	0.4324	ns	0.0027	**	0.133	ns	0.0254	*
PBS vs. galectin-3	0.0006	***	0.0084	**	0.042	*	0.0025	**
G(2) + galectin-3 vs. galectin-3	0.2292	ns	0.3425	ns	0.393	ns	0.0651	ns
G(3) + galectin-3 vs. galectin-3	0.0388	*	0.1991	ns	0.176	ns	0.1433	ns
G(4) + galectin-3 vs. galectin-3	0.0232	*	0.0666	ns	0.007	**	0.0307	*
G(6) + galectin-3 vs. galectin-3	0.0265	*	0.0154	*	0.002	**	0.0146	*

Table S16. P values for data shown in Figure 5 and S18 for HT-1080 cells. *P<0.05, **P<0.01, ***P<0.0001, ****P<0.0001, ns = not significant (P>0.05).

	2 h		4 h		6 h	
	P value	Summary	P value	Summary	P value	Summary
PBS vs. G(2)	0.6394	ns	0.6043	ns	0.285	ns
PBS vs. G(3)	0.4597	ns	0.0289	*	0.008	**
PBS vs. G(4)	0.0811	ns	0.0319	*	0.05	*
PBS vs. G(6)	0.1884	ns	0.1988	ns	0.137	ns
	8 h		10 h		12 h	
	P value	Summary	P value	Summary	P value	Summary
PBS vs. G(2)	0.796	ns	0.644	ns	0.583	ns
PBS vs. G(3)	0.1838	ns	0.008	**	0.264	ns
PBS vs. G(4)	0.0633	ns	0.1047	ns	0.1	ns
PBS vs. G(6)	0.2324	ns	0.2858	ns	0.515	ns

Table S17. P values for data shown in Figure 5 for HT-1080 cells. *P<0.05, **P<0.01, ***P<0.0001, ****P<0.0001, ns = not significant (P>0.05).

	2 h		4 h		6 h	
	P value	Summary	P value	Summary	P value	Summary
PBS vs. G(2) + galectin-3	0.071	ns	0.1117	ns	0.023	*
PBS vs. G(3) + galectin-3	0.8928	ns	0.1901	ns	0.067	ns
PBS vs. G(4) + galectin-3	0.8275	ns	0.1161	ns	0.014	*
PBS vs. G(6) + galectin-3	0.5188	ns	0.499	ns	0.041	*
PBS vs. galectin-3	0.0559	ns	0.0023	**	4E-04	***
G(2) + galectin-3 vs. galectin-3	0.9798	ns	0.2581	ns	0.036	*
G(3) + galectin-3 vs. galectin-3	0.163	ns	0.1123	ns	0.092	ns
G(4) + galectin-3 vs. galectin-3	0.0306	*	0.2227	ns	0.08	ns
G(6) + galectin-3 vs. galectin-3	0.0011	**	0.0387	*	0.077	ns
	8 h		10 h		12 h	
	P value	Summary	P value	Summary	P value	Summary
PBS vs. G(2) + galectin-3	0.0107	*	0.0707	ns	0.011	*
PBS vs. G(3) + galectin-3	0.0307	*	0.0424	*	0.008	**
PBS vs. G(4) + galectin-3	0.0103	*	0.0234	*	0.058	ns
PBS vs. G(6) + galectin-3	0.0347	*	0.0298	*	0.013	*
PBS vs. galectin-3	0.0003	***	0.0015	**	6E-04	***
G(2) + galectin-3 vs. galectin-3	0.0073	**	0.0472	*	0.007	**
G(3) + galectin-3 vs. galectin-3	0.0902	ns	0.0735	ns	0.045	*
G(4) + galectin-3 vs. galectin-3	0.0859	ns	0.0224	*	0.019	*
G(6) + galectin-3 vs. galectin-3	0.0921	ns	0.0455	*	0.033	*

Table S18. P values for data shown in Figure 7 for DU 145 cells. *P<0.05, **P<0.01, ***P<0.0001, ****P<0.0001, ns = not significant (P>0.05).

	4 h		8 h		12 h		16 h	
	P value	Summary	P value	Summary	P value	Summary	P value	Summary
galectin-3 vs. CRD	0.004	**	0.0034	**	3E-04	***	0.001	**
galectin-3 vs. PBS	0.0015	**	0.002	**	5E-04	***	0.0013	**
galectin-3 vs. G(6) + CRD	0.0187	*	0.006	**	0.001	**	0.0021	**

Table S19. P values for data shown in Figure 8 for DU 145 cells. *P<0.05, **P<0.01, ***P<0.0001, ****P<0.0001, ns = not significant (P>0.05).

	4 h		8 h		12 h		16 h	
	P value	Summary	P value	Summary	P value	Summary	P value	Summary
galectin-3 vs. PBS	0.0002	***	0.0038	**	<0.0001	****	0.0003	***
galectin-3 vs. 1xlactose + galectin-3	0.5664	ns	0.9988	ns	0.588	ns	0.6706	ns
galectin-3 vs. 3xlactose + galectin-3	0.0018	**	0.0222	*	<0.0001	****	0.0003	***
galectin-3 vs. 4xlactose + galectin-3	<0.0001	****	0.0046	**	<0.0001	****	<0.0001	****

Table S20. P values for data shown in Figure 9 for DU 145 cells. *P<0.05, **P<0.01, ***P<0.0001, ****P<0.0001, ns = not significant (P>0.05).

	P value	Summary
CRD vs. G(2)	0.9699	ns
CRD vs. G(3)	0.9921	ns
CRD vs. G(4)	0.9976	ns
CRD vs. G(6)	0.2525	ns

Table S21. P values for data shown in Figure 10 for DU 145 cells. *P<0.05, **P<0.01, ***P<0.0001, ****P<0.0001, ns = not significant (P>0.05).

	P value	Summary
G(2) + galectin-3 vs. galectin-3	0.001	**
G(3) + galectin-3 vs. galectin-3	0.0004	***
G(4) + galectin-3 vs. galectin-3	0.0005	***
G(6) + galectin-3 vs. galectin-3	<0.0001	****
G(2) + galectin-3 vs. G(3) + galectin-3	0.6447	ns
G(2) + galectin-3 vs. G(4) + galectin-3	0.833	ns
G(2) + galectin-3 vs. G(6) + galectin-3	0.0055	**
G(3) + galectin-3 vs. G(4) + galectin-3	0.9783	ns
G(3) + galectin-3 vs. G(6) + galectin-3	0.0104	*
G(4) + galectin-3 vs. G(6) + galectin-3	0.0084	**
